# Dynamic Regulation of Oct1 during Mitosis by Phosphorylation and Ubiquitination

**DOI:** 10.1371/journal.pone.0023872

**Published:** 2011-08-29

**Authors:** Jinsuk Kang, Ben Goodman, Yixian Zheng, Dean Tantin

**Affiliations:** 1 Department of Pathology, University of Utah School of Medicine, Salt Lake City, Utah, United States of America; 2 Department of Embryology, Carnegie Institution of Washington/HHMI, Baltimore, Maryland, United States of America; National Cancer Institute, United States of America

## Abstract

**Background:**

Transcription factor Oct1 regulates multiple cellular processes. It is known to be phosphorylated during the cell cycle and by stress, however the upstream kinases and downstream consequences are not well understood. One of these modified forms, phosphorylated at S335, lacks the ability to bind DNA. Other modification states besides phosphorylation have not been described.

**Methodology/Principal Findings:**

We show that Oct1 is phosphorylated at S335 in the Oct1 DNA binding domain during M-phase by the NIMA-related kinase Nek6. Phospho-Oct1 is also ubiquitinated. Phosphorylation excludes Oct1 from mitotic chromatin. Instead, Oct1^pS335^ concentrates at centrosomes, mitotic spindle poles, kinetochores and the midbody. Oct1 siRNA knockdown diminishes the signal at these locations. Both Oct1 ablation and overexpression result in abnormal mitoses. S335 is important for the overexpression phenotype, implicating this residue in mitotic regulation. Oct1 depletion causes defects in spindle morphogenesis in *Xenopus* egg extracts, establishing a mitosis-specific function of Oct1. Oct1 colocalizes with lamin B1 at the spindle poles and midbody. At the midbody, both proteins are mutually required to correctly localize the other. We show that phospho-Oct1 is modified late in mitosis by non-canonical K11-linked polyubiquitin chains. Ubiquitination requires the anaphase-promoting complex, and we further show that the anaphase-promoting complex large subunit APC1 and Oct1^pS335^ interact.

**Conclusions/Significance:**

These findings reveal mechanistic coupling between Oct1 phosphorylation and ubquitination during mitotic progression, and a role for Oct1 in mitosis.

## Introduction

The Oct1 (POU2F1) transcription factor is a potent regulator of metabolism and tumorigenicity [1]. It is widely expressed [2], [3] and interacts with a number of proteins including poly (ADP-ribose) polymerase-1 (PARP-1), an enzyme that becomes activated by DNA damage and oxidative stress [4], BRCA1, a tumor suppressor protein associated with the DNA damage response [5], [6], and lamin B, a component of the nuclear and spindle matrices [7], [8], [9]. Oct1 is also a signal integrator that is phosphorylated at multiple residues during the cell cycle and in response to genotoxic and oxidative stress [10], [11]. Some of these phosphorylation events alter Oct1 DNA binding selectivity, resulting in altered target gene occupancy [10]. Other phosphorylation events have not been carefully studied.

One of the aforementioned phosphorylation events occurs at Ser335 within the DNA binding domain. Ser335 mutation to aspartic acid blocks Oct1 binding to all tested DNA recognition sites [10], and Ser335 phosphorylation has been associated with mitosis in mass screens [12], [13], [14]. Little is otherwise known about the function of this modification. Here, we identify a previously unknown role for this form of Oct1. Consistent with the effects of S335 phosphorylation on Oct1 ability to bind DNA, we find that phosphorylation excludes Oct1 from mitotic chromosomes. Phospho-S335 Oct1 accumulates on centrosomes, spindle pole bodies and kinetochores, with enrichment lost at the anaphase-telophase transition. Late in mitosis the remaining phosphorylated Oct1 is modified by non-canonical K11-linked polyubiquitin chains and colocalizes with lamin B at the midbody. We show that the phosphorylated form of Oct1 interacts with lamin B, and that RNAi knockdown of either Oct1 or lamin B1 in HeLa cells eliminates the midbody localization of the other protein. We implicate the anaphase-promoting complex (APC) in Oct1 ubiquitination. Oct1 RNAi in HeLa cells strongly reduces antibody localization to centrosomes, spindle pole bodies and the midbody, and results in mitotic abnormalities. Overexpression of wild-type Oct1 also disrupts mitoses, resulting in improper chromosome condensation, multinucleated cells and micronuclei. Overexpressed S335A mutant Oct1 does not disrupt mitosis to the same extent, implicating phosphorylation of this residue in Oct1 regulation of mitotic functions.

## Results

### Phosphorylation of Oct1 at serine 335 during mitosis

**Figure 1:**
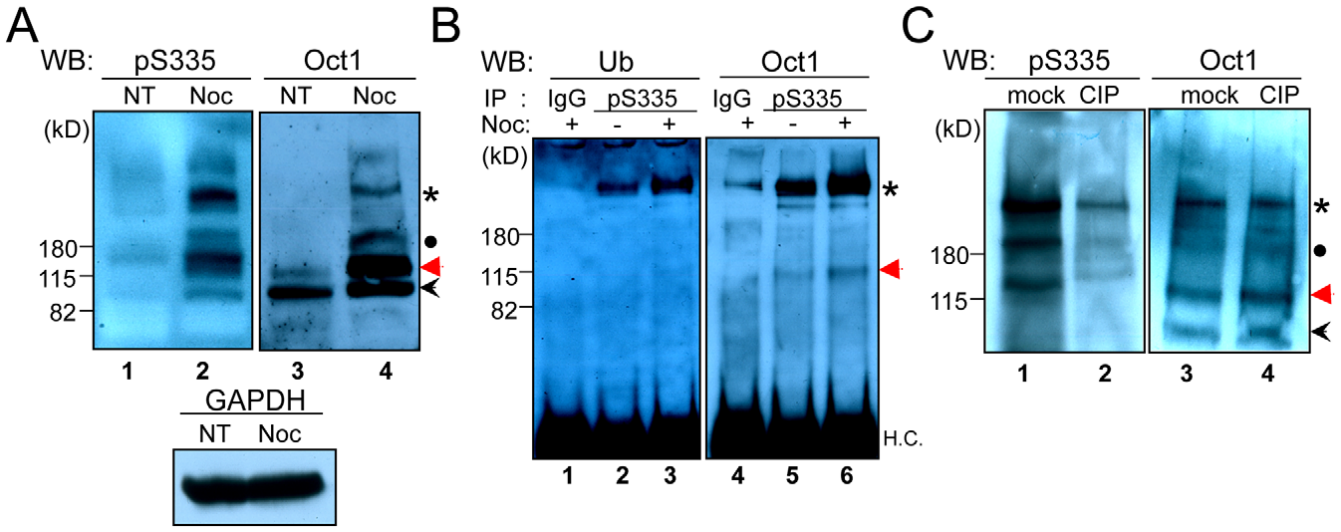
Oct1^pS335^ is enriched in M-phase HeLa cells. (**A**) Whole cell extracts were prepared from normal or nocodozole-arrested HeLa cells. 10% polyacrylamide gels were Western blotted using anti-Oct1^pS335^ or anti-pan-Oct1 (C-terminal) antibodies (Bethyl). Anti-GAPDH is shown as a loading control. (**B**) HeLa cell whole cell extracts were immunoprecipitated with anti-Oct1^pS335^ antibodies and Western blotted using pan-Ub or pan-Oct1 antibodies. H.C. = immunoglobulin heavy chain. (**C**) Whole cell extracts from nocodozole-arrested HeLa cells were treated with calf intestinal alkaline phosphatase (CIP), or mock-treated. Western blots using anti-Oct1^pS335^ or anti-pan-Oct1 antibodies are shown.

To study the regulation and function of Oct1 phosphorylation at serine 335 (Oct1^pS335^), we generated a phospho-specific polyclonal antibody. The peptide sequence used to generate the antibody (EALNLS_335_FKNMC) aligns perfectly to the POU-specific portion of the DNA binding domain of human Oct1, Oct2 and Oct11. This region is less conserved in other human POU domain proteins and does not match any other protein sequences in the non-redundant protein databases (not shown). The predicted molecular weight of unmodified human Oct1 is 80 kDa, but the standard form migrates at ∼90 kDa in denaturing SDS-polyacrylamide gels. Initial characterization of the antibody using HeLa whole cell extracts and Western blotting indicated the presence of an intense band of high apparent molecular weight (>200 kDa) in cells arrested in mitosis by nocodozole (Figure 1A, lane 2, asterisk). A band was also present in mitotic cells corresponding to the expected molecular weight of ∼90 kDa (black arrowhead), as were intermediate forms with apparent molecular weights of ∼180 kDa and ∼130 kDa (black dot and red arrow). A pan-Oct1 antibody (recognizing the C-terminus) identified the same forms, albeit at different relative intensities, with the two largest forms only found in mitotic cells (Figure 1A, lane 4). This result suggests that the four observed species are different forms of Oct1. No augmentation in Western blot signal was observed using HeLa cells arrested in S-phase with hydroxyurea (not shown), suggesting that the effects are specific to mitosis.

We hypothesized that one or more of the high molecular weight Oct1^pS335^ bands represented ubiquitinated species. To test this possibility, we immunoprecipitated Oct1 using the phospho-specific antibody and performed Western blots for both ubiquitin (Ub) and total Oct1. A band of the same size corresponding to the high molecular weight form was observed in both cases (Figure 1B, lanes 2 and 5, asterisk). The band was further enriched using extracts from nocodozole arrested cells (lanes 3 and 6), indicating the presence of an Oct1 population enriched in mitotic cells that is simultaneously phosphorylated at S335 and ubiquitinated. A similar result was obtained using denaturing conditions, indicating that the phospho-specific antibody is not co-precipitating a ubiquitinated protein of precisely the same apparent molecular weight, but rather recognizes a phosphorylated, ubiquitinated form of Oct1. These experiments also revealed that the high molecular weight form of Oct1 consists of a ladder of bands (Figure S1A).

To demonstrate that the form of Oct1 recognized by the antibody is phosphorylated, we treated nocodozole-arrested HeLa cell extracts with calf intestinal alkaline phosphatase (CIP), which resulted in the strong diminution of the high molecular weight form, as well as other Oct1 forms, but had no effect on total Oct1 (Figure 1C). No increase in band mobility was observed with the pan-Oct1 antibody following CIP treatment, consistent with the finding that that ubiquitination is also present on Oct1^pS335^. Similar loss of phospho-specific antibody signal with CIP treatment was also observed using indirect immunofluorescence (IF) assays (Figure S1B).

**Figure 2:**
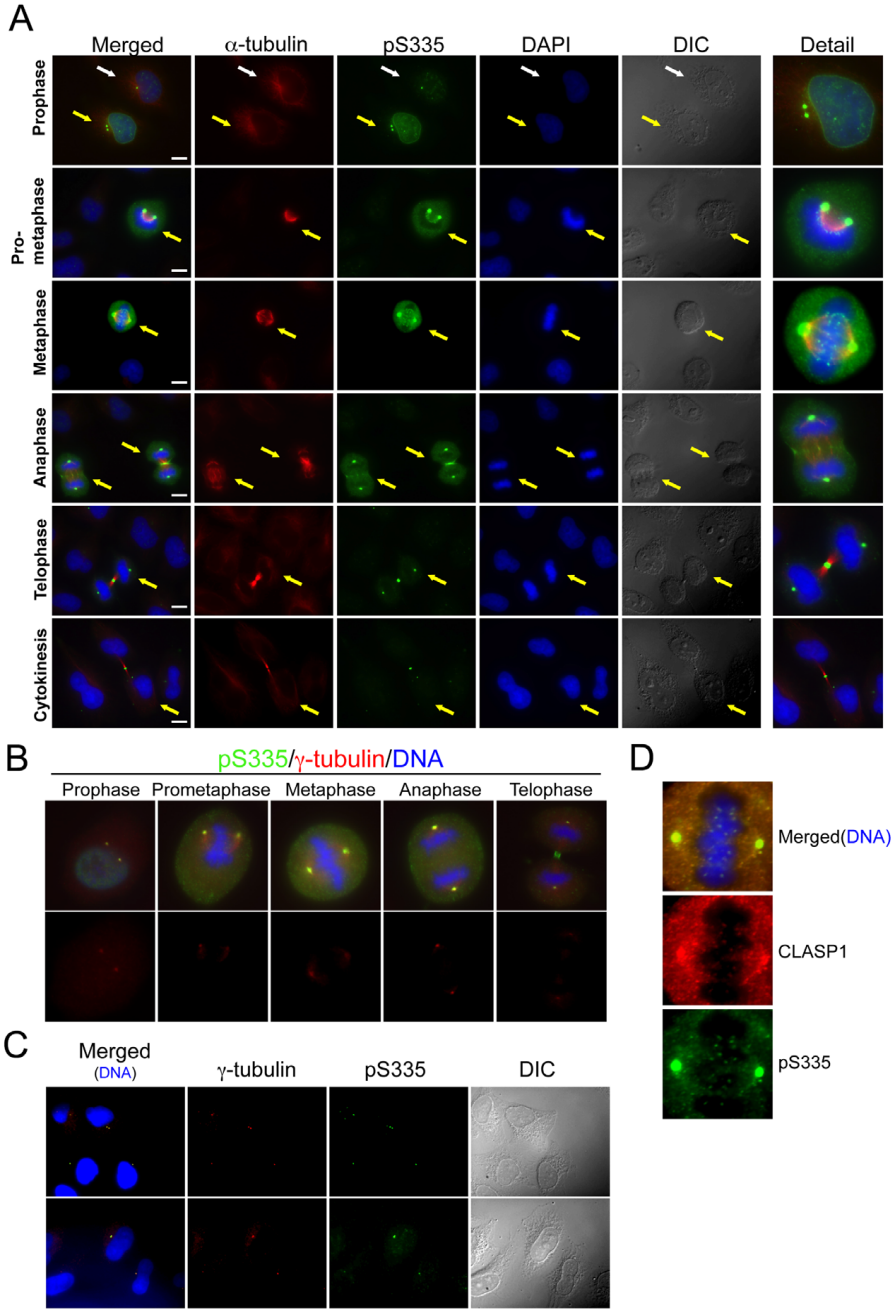
Mitotic Oct1^pS335^ is associated with the spindle pole bodies and midbody. (**A**) IF images of mitotic HeLa cells are shown. Cells were stained with anti-α-tubulin and anti-Oct1^pS335^ antibodies, and with DAPI. Scale bar: 20 µM. White arrows show an interphase cell. Yellow arrows show different mitotic stages. (**B**) Similar images, except γ-tubulin antibodies were used. Cropped images of individual mitotic cells are shown. (**C**) Similar images of interphase cells. (**D**) Detail of a metaphase HeLa cell IF image stained with CLASP-1 and Oct1^pS335^ antibodies.

We examined phosphorylated Oct1 in HeLa cells using IF and the anti-Oct1^pS335^ antibody (Figure 2A). HeLa mitoses were staged using DAPI and anti-α-tubulin. Oct1^pS335^ staining in interphase cells (Figure 2A, white arrows) was largely confined to individual puncta, the most intense of which correspond to the site of microtubule nucleation. Increased Oct1^pS335^ signal at an intact nuclear envelope was noted in prophase (yellow arrows). Early mitotic cells also showed staining at two puncta suggestive of duplicated centrosomes. Oct1^pS335^ was enriched at the prophase nuclear envelope (see prophase detail). Work of others has associated Oct1 with the nuclear periphery [7], [8], [15]. At metaphase, the chromosomes become fully condensed, and Oct1^pS335^ was largely excluded from DNA, except at small puncta consistent with kinetochores. We corroborated spindle pole body and centrosome localization in mitotic and interphase cells using γ-tubulin antibodies (Figure 2B and C), and kinetochore localization using CLASP1 antibodies (Figure 2D).

Following chromatid separation, Oct1^pS335^ becomes concentrated at the developing midzone/contractile ring in anaphase and later in telphase at the midbody (Figure 2A). At the transition from late anaphase to telophase, detectable Oct1^pS335^ was greatly diminished with the exception of the midbody, where a concentrated signal was retained throughout cytokinesis. No such change was detected using pan-Oct1 antibodies (Figure S2), indicating that the diminution of the phosphorylated form at the anaphase-telophase transition is not the result of changes in total Oct1. IF using pan-Oct1 antibodies showed similar concentrations at spindle pole bodies and the midbody (Figure S2). Similar mitotic staining patterns were also obtained using A549 lung adenocarcinoma cells (Figure S3), indicating that the pattern is not peculiar to HeLa cells. A comparative analysis of Oct1^pS335^ and the well-established mitotic marker histone H3^pS10^ [16] indicated that all cells that stained for histone H3^p10^ also stained strongly for Oct1^pS335^ (Figure S4).

### Oct1 ablation is associated with abnormal mitoses

**Figure 3:**
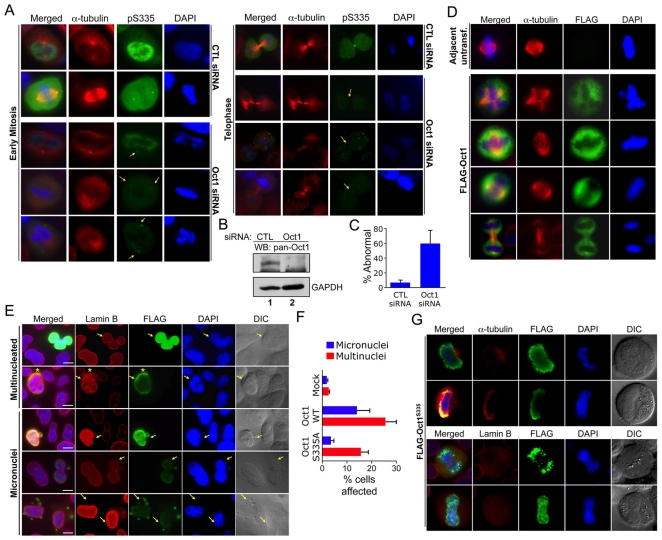
Modulation of Oct1 levels results in abnormal mitoses in HeLa cells. (**A**) IF images are shown of HeLa cells transiently transfected with control or Oct1-specific siRNAs. Arrows indicate positions of disrupted spindle pole/midbody localization. Formaldehyde fixation was used. (**B**) Western blot showing effect of transfected control and Oct1-specific siRNA on total Oct1 expression in cycling cells. Cells were cultured for 72 hr prior to analysis. (**C**) Quantification of abnormal in HeLa cells treated with control and Oct1-specific siRNAs. Values represent averages from three independent experiments. Error bars depict standard deviations. (**D**) IF images of HeLa cells transiently transfected with FLAG-Oct1. Transfected cells were incubated for 48 hr prior to formaldehyde fixation and staining with anti-α-tubulin and anti-FLAG antibodies. Single mitotic cell images are shown. (**E**) IF images are shown of interphase HeLa cells transiently transfected with FLAG-tagged wild-type Oct1. Cells were stained with DAPI, and anti-FLAG and anti-lamin B (B1+B2) antibodies. Arrows indicate transfected cells. Asterisk indicates an area of specific Oct1 and lamin B co-localization. Example cells showing multinucleated cells and micronuclei are shown. Scale bars indicate 20 µM. (**F**) Quantification of the frequency of micronuclei and multinucleated cells in mock transfected, wild-type Oct1 transfected, and S335A transfected interphase HeLa cells. Error bars depict standard deviations. (**G**) Similar experiment as (E) using cells transiently transfected with an Oct1 S335A point mutant made using site-directed mutagenesis of the human cDNA. Top panels show mitotic HeLa cells stained with anti-FLAG and anti-α-tubulin antibodies. Bottom panels substituted lamin B antibodies.

We transiently transfected HeLa cells with Oct1 siRNAs, analyzing cells 48 hr post-transfection to determine whether the staining patterns were specifically due to Oct1. Using Oct1-specific but not control siRNAs , we observed mitotic cells lacking or with significantly reduced Oct1^pS335^ spindle pole body/midbody staining (Figure 3A). In addition, nearly all (>90%) of the mitotic cells with decreased Oct1 staining lost the normal pattern of α-tubulin staining (Figure 3A). We confirmed the effect of transiently transfected Oct1-specific siRNAs by Western blot (Figure 3B). We also noted poor/abnormal chromosome segregation and other mitotic irregularities associated with partial and complete Oct1 knockdown (Figure 3A and Figure S5). To quantify these irregularities, we analyzed 668 total control siRNA and 164 total Oct1-specific siRNA mitoses over three separate experiments. There were fewer mitotic events in the Oct1-specific siRNA condition and more dead/floating cells, suggesting that following acute reductions in Oct1, a higher fraction of mitoses result in apoptosis and/or mitotic catastrophe. We scored approximately 10% of live control mitotic events as abnormal, based on spindle disorganization and chromosomal abnormalities (e.g., incomplete condensation, DNA outside the metaphase plate). Using the same criteria, approximately 60% of the Oct1 knockdown events were abnormal (Figure 3C). These findings indicate that Oct1 contributes to accurate mitosis in HeLa cells.

We also examined mitoses in Oct1 deficient primary early-passage murine fibroblasts. Oct1 protein and activity is undetectable in these cells in Western blotting and gel mobility shift assays using nuclear extracts, although a small amount of residual protein is observed upon enrichment by affinity purification [17]. No IF signal was detected in Oct1 deficient MEFs using pan-Oct1 antibodies (not shown). Using the phospho-specific antibody, wild-type fibroblasts displayed a similar, albeit less uniform, mitotic staining pattern as HeLa cells. The signal was diminished but not eliminated in *Oct1^−/−^* fibroblasts (Figure S6). These results suggested the presence of a cross-reacting co-expressed POU transcription factor in fibroblasts. Oct1 is the sole detectable octamer DNA binding activity in MEFs [17]. We therefore focused on non-octamer binding POU transcription factors with capacity to cross-react. One murine protein, Pit-1/POU1F1, contains a perfect match to the peptide sequence used to generate the phospho-specific antibody. Western blotting using pan-Pit-1 antibodies indicated that Pit-1 was expressed in murine fibroblasts but not HeLa cells (Figure S6). Either due to redundancy with Pit-1 or other differences between HeLa cells and primary murine fibroblasts, we observed more mild evidence of abnormal mitoses in *Oct1^−/−^* fibroblasts, including occasional abnormal DNA condensation and abnormal spindles. Analysis of DNA content also revealed the presence of aneuploidy, and an increase in cells with >4N DNA in *Oct1^−/−^* fibroblasts (Figure S6).

### Overexpression of Oct1 results in abnormal mitoses

We tested the effect of overexpressed full-length FLAG-tagged wild-type and S335A Oct1 in HeLa cells using transient transfection. Overexpression of wild-type protein resulted in significantly increased Oct1^pS335^ staining, in particular the generation of interphase Oct1^pS335^–staining puncta, while little change was observed using the S335A mutant (Figure S7). In mitotic cells, overexpressed wild-type FLAG-Oct1 was also excluded from mitotic chromatin and resulted in disorganized mitotic microtubules as compared to adjacent untransfected controls (Figure 3D). We also studied interphase cells, using lamin B (B1+B2) antibodies to visualize the nuclear envelope. Adjacent untransfected (FLAG-negative) HeLa cells served as an internal control. Cells in which Oct1 was concentrated in particular areas also displayed lamin B concentrations in the same areas (Figure 3E, asterisks), consistent with a described interaction between the two proteins [7], [8], [9]. Interphase cells overexpressing FLAG-Oct1 displayed increases in multinucleation and micronuclei. The micronuclei contained FLAG-Oct1 (Figure 3E, and Figure S8). S335A Mutant Oct1 was incapable of inducing micronuclei, and displayed reduced capacity to induce multinucleated cells (Figure 3F). Unlike wild-type Oct1, overexpressed S335A mutant Oct1 also could be found at mitotic DNA (Figure 3G). This result indicates that S335 is required for exclusion from mitotic chromatin.

### Nek6 phosphorylates Oct1 serine 335 during mitosis

**Figure 4:**
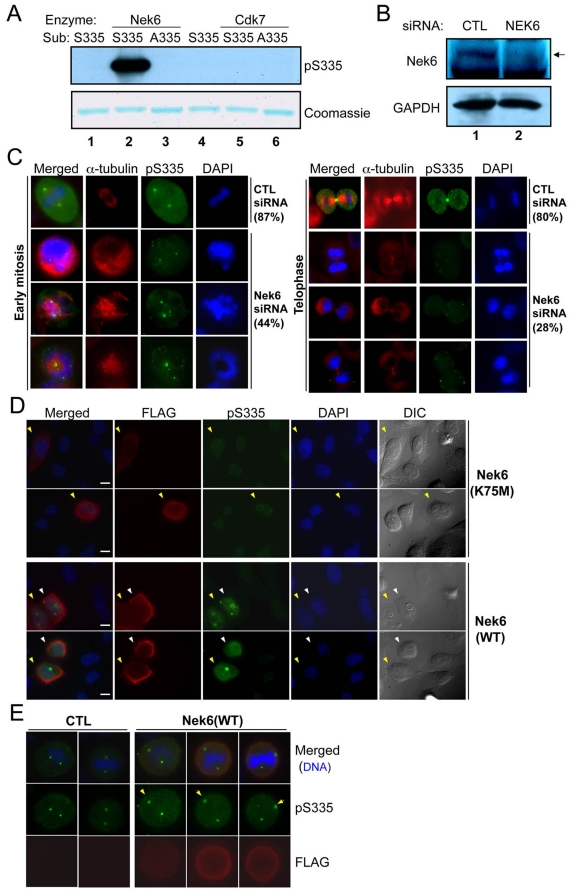
Nek6 contributes to mitotic Oct1 phosphorylation at S335. (**A**) In vitro kinase assay using purified recombinant Nek6 or Cdk7, and GST fused to wild-type or mutant Ser335 target peptide sequences. A Coomassie blue-stained SDS-polyacrylamide gel is also shown to confirm presence of the purified peptide. (**B**) Nek6 knockdown in HeLa cells. A Western blot using anti-Nek6-specific antibodies is shown. Extracts were prepared 72 hr post-transfection. (**C**) HeLa cells were transfected with scrambled and Nek6-specific siRNAs, incubated for 72 hr, fixed and stained with DAPI, anti-α-tubulin and anti-Oct1^pS335^ antibodies. Examples of early (left) and late (right) mitoses are shown. Early mitotic percentages reflect the number of events showing strong Oct1^pS335^ staining (42/48 in the control vs. 21/47 in the Nek6 specific knockdown). Telophase percentages reflect the number of events showing strong midbody staining (33/41 vs. 11/39). Formaldehyde fixation was used. (**D**) HeLa cells were transiently transfected with FLAG-tagged wild-type Nek6 or catalytically inactive mutants (K75M), incubated for 24 hr, and prepared as in (B). Examples of interphase cells are shown. Arrows indicate transfected (FLAG-positive) cells. Where two (yellow and white) arrows are present, two adjacent transfected cells are shown. Formaldehyde fixation was used. (**E**) Mitotic examples. Arrows indicate examples of spindle poles that are more strongly stained with anti-phospho-Oct1 when Nek6 is over-expressed. Formaldehyde fixation was used.

Computational inspection of Oct1 using the phosphorylation site database PHOSIDA (http://www.phosida.com/) identified a consensus Nek6 kinase target site at S335. Nek6 is a NIMA-related kinase required for normal mitosis in HeLa cells [18]. We tested the ability of recombinant purified Nek6 to phosphorylate an Oct1 peptide containing S335 fused to recombinant glutathione S-transferase (GST) in vitro. GST-peptide fusions with a mutated target serine residue and a different kinase (Cdk7) were used as controls. Nek6 but not Cdk7 robustly produced a reactive target peptide, but generated no signal using mutant GST-fused peptides (Figure 4A). We repeated these experiments using radiolabeled ATP to demonstrate that Nek6 was not phosphorylating the peptide at another position, and to show that full-length recombinant Oct1 was phosphorylated by Nek6 (Figure S9). The data are consistent with a model in which Nek6 phosphorylates Oct1 during mitosis. To test this model, we transiently transfected Nek6-specific and scrambled control siRNAs into HeLa cells. At 72 hr post-transfection the knockdown was robust (Figure 4B). We focused on mitotic events (rounded cells with duplicated centrosomes and partially/fully condensed chromosomes). In early mitosis, Nek6 knockdown reduced the intensity and uniformity of Oct1^pS335^ staining and disrupted the organization of mitotic spindles (Figure 4C, left panels). Later in mitosis, Nek6 knockdown also reduced the overall staining intensity, and specifically ablated Oct1^pS335^ detected at the midbody (right panels). We quantified the degree of pan-Oct1 or Oct1^pS335^ signal intensity in all mitotic cells following control or Nek6 siRNA transfection, observing an overall two-fold decrease in Oct1^pS335^ but not pan-Oct1 in Nek6 but not control siRNA (Figure S9). We overexpressed FLAG-tagged wild-type or catalytically inactive mutant Nek6 [18] (plasmids a gift of A. Fry) to determine the effect on Oct1 phosphorylation. Overexpression of wild-type but not mutant Nek6 resulted in accumulation of diffuse Oct1^pS335^ and brighter Oct1^pS335^ puncta localizing to interphase centrosomes (Figure 4D). Moreover, additional Oct1^pS335^ puncta in interphase, and mis-localization of mitotic spindle poles, were observed. Mitotic HeLa cells also showed increased Oct1^pS335^, although the baseline expression was higher (Figure 4E). These results indicate that Oct1 S335 is a Nek6 target.

### Oct1 is a component of the spindle matrix and participates in a complex with lamin B1 at the midbody

**Figure 5:**
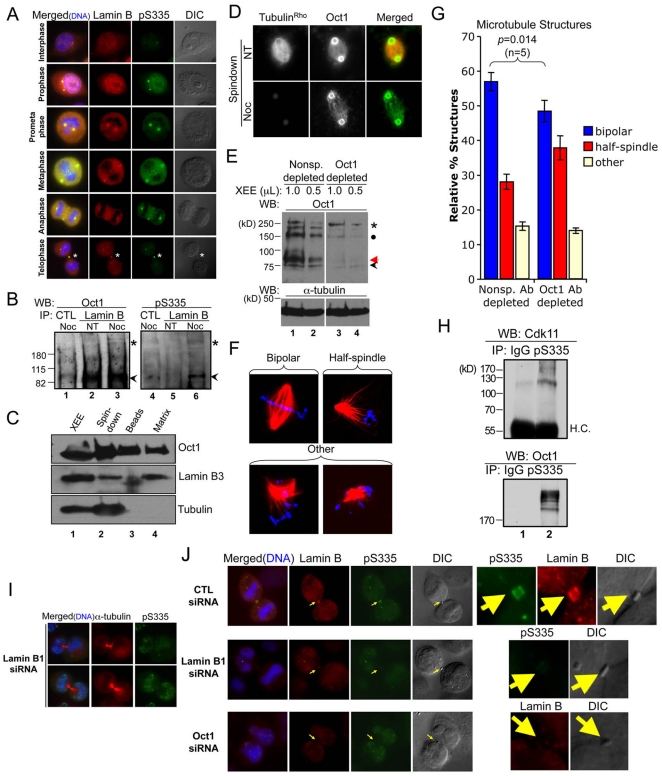
Oct1 is present in the spindle matrix and forms a complex with lamin B1 at the midbody in HeLa cells. (**A**) Association of phosphorylated Oct1 with lamin B at the centrosomes and midbody. HeLa cells were fixed and stained with antibodies against lamin B1+B2 and Oct1^pS335^. Mitotic stage is on the left. Asterisk indicates the midbody structure. (**B**) Whole cell extracts from cycling HeLa cells and cells arrested in M-phase using nocodozole were immunoprecipitated using mouse anti-lamin B antibodies. Left panel shows a Western blot using pan-Oct1 antibodies. Black arrow shows predicted Oct1 molecular weight. Asterisk shows the high molecular weight form identified in Fig. 1. Right panel: the blot was stripped and re-probed using Oct1^pS335^ antibodies. (**C**) Spindle matrix preparations generated from *Xenopus* oocyte extracts (XEE, lane 1) were Western blotted using pan-Oct1, lamin B3, and α-tubulin antibodies. (**D**) IF images of bead spindown preparation. Pan-Oct1 antibodies, and rhodamine-conjugated α-tubulin were used. (**E**) *Xenopus* Oct1 was immunodepleted using magnetic protein A-coupled beads (see methods). Oct1 Western blots are shown of the non-specific and Oct1-specific depletions. α-tubulin is shown as a loading control. (**F**) Examples of spindle structures generated using the depleted extracts. Images of structures conforming to the scoring criteria used in (G) are shown. (**G**) Quantification of spindle structures using non-specific of Oct1-specific depletion. Error bars depict standard error of the mean. (**H**) Co-immunoprecipitation Cdk11 with endogenous phospho-Oct1. Mitotic-arrested HeLa whole cell extracts were immunprecipitated using phospho-Oct1 antibodies and probed with anti-Cdk11 or anti-pan-Oct1. Arrest was accomplished with 18 hr treatment with nocodozole. (**I**) HeLa cells were transiently transfected with Lamin B1-specific siRNAs. Cells were incubated for 72 hr, fixed and stained with α-tubulin and pS335 antibodies. Images of cells undergoing abcission are shown. Formaldehyde fixation was used. (**J**) HeLa cells transfected with control siRNAs, or siRNAs against Oct1 or lamin B1 were fixed and stained with lamin B and Oct1^pS335^ antibodies. IF images of mitotic HeLa cells undergoing abcission are shown. Arrow indicates position of the midbody. Detail at right shows isolated midbody structures. Formaldehyde fixation was used.

Our results suggested that Oct1 recruitment to mitotic structures is important for normal mitoses. We therefore attempted to identify whether other proteins known to interact with Oct1 recruit it to these structures. Lamin B has been shown to interact with Oct1 and can co-localize with Oct1 at the nuclear envelope [7], [8]. Lamin B is also present at a structure known as the spindle matrix, and is required for proper spindle organization [19]. We used antibodies against total lamin B1+B2 to observe localization in HeLa mitoses. We observed substantial co-localization between lamin B and Oct1^pS335^. In particular, the spindle poles and midbody were strongly stained with both proteins (Figure 5A, asterisks).

To determine whether phosphorylated Oct1 and lamin B interact, we performed co-immumoprecipitation experiments using whole cell extracts from untreated or nocodozole-arrested HeLa cells. As expected, immunoprecipitation with lamin B antibodies enriched total Oct1 in cycling HeLa cells (Figure 5B, lane 2, arrow). Equivalent enrichment was also observed M-phase arrested cells (lane 3), indicating that even after nuclear envelope breakdown the interaction between Oct1 and lamin B is preserved. These data indicate that the known interaction between Oct1 and lamin B1 can be extended to mitosis. Immunobloting using Oct1^pS335^ antibodies also uncovered an interaction between mitotic phosphorylated Oct1 and lamin B 9 (lane 6, arrow). The high molecular weight form of Oct1 enriched in mitosis interacted only poorly with lamin B (lane 6, asterisk).

Prior studies in *Xenopus* have shown that lamin B helps to form a network referred to as the spindle matrix during mitosis. This structure associates with the spindle to help maintain spindle pole focus and spindle shape. Mass spectrometry analyses revealed that a number of transcription factors including Oct2, an Oct1 paralog, are present in isolated spindle matrix [19], [20]. Spindle matrix components can be isolated from *Xenopus* egg extracts (XEE) using a spindle assembly assay stimulated by magnetic beads coated with the mitotic kinase Aurora A [19], [20]. The beads function as potent microtubule nucleating and organizing centers and efficiently organize spindle poles. We retrieved the bead-associated spindles using a magnet (Figure 5C, “Spindown”). Buffer containing nocodozole was used to depolymerize spindle microtubules. The beads (Figure 5C, lane 3) and their associated spindle matrix (lane 4) were separated from each other. We identified a band corresponding to Oct1 in the spindle, beads, and matrix preparations using pan-Oct1 antibodies. As expected, most lamin B3, the major form of lamin B in XEE, was present in the spindle matrix (Figure 5C, lane 4). The presence of Oct1 in the beads and the spindle matrix is consistent with the idea that subsets of Oct1 are associated with the spindle poles and surrounding matrix. We visualized the bead preparations and the associated matrix using fluorescence microscopy. Robust levels of Oct1 associated with the beads themselves as well as the associated matrix (Figure 5D).

The above result suggested that loss of *Xenopus* Oct1 could have functional effects in this assay, although there is a distinct possibility of redundancy with the Oct2 protein. To test this prediction, we immunodepleted Oct1 from XEE and reconstituted the assay. Overall Oct1 immunodepletion was estimated at ∼85% based on band intensity, although the antibody showed some variation in the ability to immunodeplete different Oct1 species (Figure 5E). Immunodepleted extracts successfully reconstituted mitotic spindle structures, however there was a statistically significant defect in ability to form normal bipolar spindle structures relative to mock-depleted extracts, and a corresponding increase in aberrant monopolar structures (Figure 5F–G).

We expressed Oct1 fused to GFP in MEFs and used anti-GFP coupled beads to identify associated proteins via mass spectrometry (unpublished data). One of the identified proteins was Cdk11 (not shown), which regulates mitotic spindle stability. Cdk11 depletion results in similar in vitro defects in spindle assembly [21], suggesting that Cdk11 co-depletion may contribute to the phenotype. We therefore performed co-immunoprecipitation with endogenous Oct1 and Cdk11 to confirm this interaction. Whole cell extracts from nocodozole-arrested HeLa cells were precipitated using anti-Oct1^pS335^ and Western blotted using Cdk11 and control pan-Oct1 antibodies (Figure 5H). The results identify an interaction with the known mitotic regulator Cdk11 and Oct1, confirming the mass spectrometry and extending the interaction to the native protein.

We next silenced lamin B1 using siRNAs and monitored Oct1^pS335^ localization during metaphase and anaphase. Lamin B1 silencing did not change Oct1 protein levels, but resulted in early mitotic defects such as mitotic spindle disruption and unfocused spindle poles (Figure S10), as reported previously [19]. Phospho-Oct1 localization to both kinetochores and spindle poles remained intact, suggesting that lamin B1 is not required for Oct1 localization to these two structures. However, interestingly in late mitosis Oct1 midbody localization was abolished (Figure 5I). This result suggested that lamin B1 localizes phosphorylated Oct1 to the midbody. To study the interaction of Oct1 and lamin B at the midbody, we knocked down Oct1 and lamin B1 in HeLa cells, and stained with lamin B (B1+B2) and Oct1^pS335^ antibodies. Control-siRNA transfected HeLa cells showed expected concentrations of lamin B and Oct1^pS335^ at the midbody (Figure 5J, top panel and detail at right). Due to the presence of lamin B2, total lamin B staining was minimally affected, however lamin B concentration at the midbody was largely eliminated (Figure 5J, middle panels), indicating that midbody lamin B consists mostly of lamin B1 in HeLa cells. In the lamin B1 knockdown condition, Oct1^pS335^ midbody staining was also absent in 50% of cells undergoing cytokinesis and significantly depleted in others (detail at right), suggesting that lamin B1 recruits phospho-Oct1. Oct1 knockdown (lower panels) also quantitatively depleted lamin B1+B2 at the midbody. These findings indicate that during cytokinesis lamin B1 and Oct1 are mutually required for midbody localization.

### Mitotic Oct1^pS335^ is modified by K11-linked poly-Ub chains associated with the midbody

**Figure 6:**
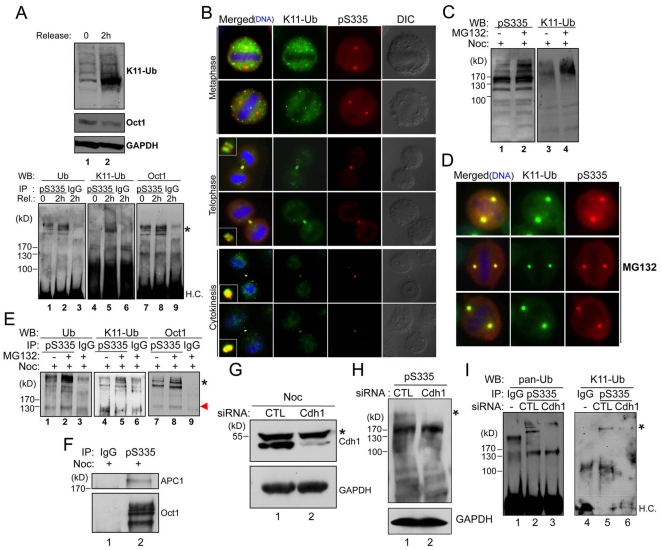
Dynamic ubiquitination of Oct1 during mitosis. (**A**) HeLa cells were blocked in G1 with thymidine, released and arrested in mitosis using 0.1 µM nocodozole as described [22]. Top panels show Western blot with K11-Ub and pan-Oct1 antibodies, and GAPDH antibodies as a loading control, from cells arrested with nocozole or following 2 hr release from nocozole. For the bottom panels, samples were immunoprecipitated using anti-Oct1^pS335^ antibodies, and probed with antibodies against pan-Ub, K11-Ub and pan-Oct1. H.C. = immunoglobulin heavy chain. (**B**) IF images of HeLa mitoses stained with Oct1^pS335^ and K11-Ub antibodies. Two metaphase, telophase and late cytokinesis examples are shown. Merged images from the two latter cases also show detail of the midbody structure (inset). (**C**) HeLa cells were arrested with nocodozole (0.5 µM) for 18 hr, and then incubated with MG132 for a further 6 hr. Whole cell extracts were prepared and subjected to Western blotting using Oct1^pS335^ and K11-Ub antibodies. (**D**) HeLa cells were treated with MG132, fixed and subjected to IF using K11-Ub and Oct1^pS335^ antibodies. Detail of metaphase cells is shown. (**E**) Whole cell extracts from HeLa cells arrested as above were immunoprecipitated with anti-Oct1^pS335^ antibodies and Western blotted using pan-Ub, K11-Ub or pan-Oct1 antibodies. H.C. = immunoglobulin heavy chain. (**F**) Nocodozole-arrested HeLa whole cell extracts were immunoprecipitated using Oct1^pS335^ antibodies and probed using pan-Oct1 or APC1 (AbCam). (**G**) Verification of Cdh1 knockdown. HeLa cells were transfected with Cdh1 siRNAs for 48 hr, after which cells were treated with 0.5 mM nocodozole for 18 hr. A Western blot is shown using Cdh1 antibodies. GAPDH is shown as a loading control. (**H**) HeLa cells were transfected with Cdh1 siRNAs. 24 hr-post transfection, cells were treated with 0.5 µM nocodozole. 42 hr-post transfection, cells were treated with MG132 for 6 hr. Whole cell extracts were prepared after 48 hr. (**I**) siRNA-transfected HeLa cells as in (H) were immunoprecipitated using Oct1^pS335^ antibodies and Western blotted using pan-Ub or K11-Ub antibodies.

Our data suggested that there are qualitative differences between Oct1^pS335^ midbody localization and localization to other mitotic structures: midbody association is maintained after most signal is eliminated (Figure 2A), and lamin B is required for Oct1^pS335^ midbody localization but not localization to other structures (Figure 5F–H). We therefore sought biochemical correlates that may underlie these differences. Proteins modified by non-canonical K11-linked poly-Ub chains are enriched in the midbody [22]. We tested whether Oct1 is modified through K11-linked poly-Ub chains (K11-Ub) using a specific antibody [22]. Although Oct1 is ubiquitinated in cycling or nocodozole-arrested HeLa cells (Figure 1C), immunoprecipitation of phospho-Oct1 from these cells and Western blotting using K11-Ub antibodies produced little evidence of Oct1 K11-linked ubiquitination (not shown). K11 accumulates late in mitosis [22] suggesting that Oct1 may be modified by canonical Ub early in mitosis, but switches to a K11-linked form at later stages. We therefore arrested cells in G1/S with thymidine, released them from the thymidine block and arrested them in mitosis with nocodozole. Following release from nocodozole arrest, K11-Ub linkages were detectable after two hours (Figure 6A). Under the same conditions, immunoprecipitated Oct1^pS335^ was associated with K11-Ub as assessed by Western blot (Figure 6A, lower panels). To confirm this finding, we co-stained HeLa cells with the Oct1^pS335^ and K11-Ub antibodies and examined late stage mitoses (Figure 6B). In metaphase, both K11-Ub and Oct1^pS335^ were excluded from mitotic chromatin, however the two antibody signals displayed little overlap. For example, no K11-Ub was detected in the spindle poles. Telophase cells showed co-localization to the developing midbody, however the two signals remained spatially distinct, with K11-Ub flanking the more centrally localized Oct1^pS335^ (Figure 6B, see inset detail at left). In contrast, cells late in cytokinesis showed tight spatial overlap at the midbody.

The above results are consistent with models in which Oct1 K11-Ub occurs exclusively late in mitosis, or in which Oct1 is continually modified but is rapidly degraded, with degradation slowing or ending at late mitosis. To distinguish these two possibilities, we treated 24 hr nocodozole-arrested HeLa cells with the proteosome inhibitor MG132 during the final 6 hr. Nocodozole-arrested cells showed some evidence of Oct1^pS335^ K11-Ub (Figure 6C, lane 1), presumably because the nocodozole arrest was less precise than that in Figure 6A using a thymidine block. As expected, MG132 treatment caused total K11-Ub-modified proteins to accumulate (lane 4). In addition, the higher molecular weight forms of phosphorylated Oct1 were increased while the lower molecular weight forms were unaffected (Figure 6C, lane 2). These results indicate that Oct1 ubiquitinated species can be enriched by proteasome inhibition. Using IF, we found that mitotic HeLa cells treated with MG132 showed K11-Ub colocalization with Oct1^pS335^ at the spindle poles (Figure 6D). Mitotic cells treated with MG132 also showed larger spindle pole puncta (compare Figure 6D with Figure 6B). We therefore immunoprecipitated Oct1^pS335^ from extracts of nocodozole/MG132-treated HeLa cells. Treatment with MG132 increased total and K11-specific Oct1^pS335^ ubiquitination (Figure 6E, lanes 2 and 5). These results are consistent with a model in which Oct1^pS335^ undergoes cycles of K11 ubiquitination and destruction throughout mitosis, except at later stages when the protein is stabilized and detectable.

One activity known to catalyze K11-linked ubiquitination is the anaphase promoting complex/cyclosome (APC/C) [22], [23], [24]. To test whether Oct1 and APC/C interact, we performed co-immunoprecipitation using anti-Oct1^pS335^ and extracts from mitotic HeLa cells. Western blotting revealed the presence of not only Oct1 but also the APC/C large subunit APC1 in the immunoprecipitate (Figure 6F). To demonstrate a causal connection, we knocked down *Fzr1*, which encodes the APC/C component Cdh1, using siRNAs [22]. Fzr1 mRNA (not shown) and APC/C^Cdh1^ protein (Figure 6G) was efficiently ablated in mitotic-arrested HeLa cells. Specific but not control siRNA transfection significantly attenuated the high molecular weight phosphorylated form of Oct1 (Figure 6H). Further, Cdh1 knockdown attenuated several ubiquitinated Oct1^pS335^ forms in nocodozole-arrested, MG132-treated HeLa cells, as measured by Oct1^pS335^ immunoprecipitation followed by Western blotting with pan-Ub or K11-Ub antibodies (Figure 6I, lane 3), including a K11-Ub-modified species (lane 6). The degree of specific K11-Ub reduction averaged 37% in these experiments (not shown). Lastly, Cdh1 knockdown eliminated the ability of MG132 to redistribute K11-Ub signal to sites of Oct1 phosphorylation, implicating the APC in the deposition of K11-Ub at phosphorylated Oct1 that can be visualized when proteasome degradation is blocked (Figure S11).

## Discussion

Here we show that Oct1 phosphorylated at position S335 by Nek6 is ubiquitinated and associates with mitotic structures. Oct1^pS335^ is displaced from mitotic chromatin and concentrated at spindle pole bodies and the midbody during mitosis. Interphase cells show Oct1^pS335^ staining at centrosomes. The signal detected during mitosis is qualitatively different from that observed during interphase, and cannot be explained simply by increased total Oct1 levels. With the exception of the midbody, Oct1^pS335^ staining is rapidly lost at the anaphase-telophase transition. We identify Nek6 is an upstream Oct1^pS335^ kinase. Previous studies have shown that Nek6 localizes to the centrosomes in interphase cells, and to the spindle poles and midbody during mitosis. Nek6 loss of function also results in mitotic abnormalities and apoptosis in HeLa cells [18]. The activities of Oct1-associated proteins are consistent with these findings. For example, the DNA damage sensing factor BRCA1 is known to interact with Oct1 [5], [6] and is a known mitotic regulator that localizes to centrosomes [25], [26], [27]. PARP-1 also interacts with Oct1 [4] and localizes to centrosomes. PARP-1 is important for centrosomal function, including limiting their duplication [28].

Oct1 phosphorylation has been investigated previously within the context of the cell cycle. Mitotic phosphorylation was described at a different residue, S385 [11]. S385 phosphorylation was found to be cell cycle dependent and mediated by PKA. It was also noted that Oct1 purified from M-phase cells did not bind to DNA [29]. Later it was shown that Oct1 is excluded from mitotic chromatin [30]. Recent screens [12], [13], [14] identified enrichment in both Oct1 S335 and S385 phosphorylation during M-phase. We found that phosphorylation at S385 does not block DNA binding but instead alters the Oct1 selectivity for different DNA binding configurations [10]. We postulate that S385 phosphorylation correlates with S335 phosphorylation in mitosis, but that it is S335 phosphorylation that causes Oct1 exclusion from mitotic chromosomes. We substantiated this hypothesis by showing that overexpressed FLAG-tagged Oct1 is excluded from mitotic DNA while Oct1 with a S335A mutation is not.

**Figure 7:**
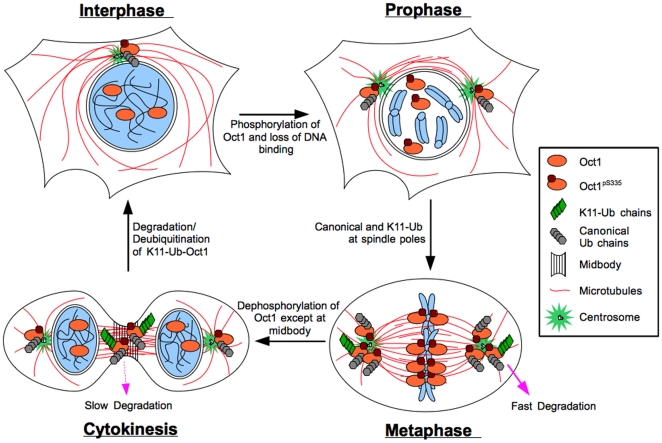
Model for Oct1 localization and modification through the cell cycle. Oct1 occupies sites in the DNA and regulates gene expression during interphase. Oct1^pS335^ localizes to centrosomes. Early in mitosis Oct1 is phosphorylated by Nek6 and localizes to spindle pole bodies and kinetochores. Oct1 is also ubiquitinated. Oct1 modified through non-canonical K11-linked Ub chains is rapidly degraded by the proteasome and is not readily detectable unless degradation by the proteasome is inhibited. Late in mitosis the bulk of phosphorylated Oct1 is de-phosphorylated, with the remaining phosphorylated Oct1 concentrated at the midbody. K11-Ub is readily detectable at the midbody, presumably because degradation has slowed or stopped. Following abcission the remaining phosphorylated Oct1 is de-phosphorylated, degraded or relocated to the centrosome.

Oct1^pS335^ is also ubiquitinated, including by non-canonical K11-Ub late in mitosis. Ubiquitinated proteins have been previously associated with the spindle pole bodies and midbody [31], [32]. Although we found that K11-Ub-modified Oct1 was detectable only in late mitoses in normal HeLa cells, cells treated with the proteasome inhibitor MG132 accumulated K11-Ub at structures to which Oct1 localizes in early mitotic stages. This result suggests a model in which K11-Ub modified Oct1^pS335^ is formed throughout mitosis, but is degraded rapidly prior to telophase, at which time degradation slows or stops. A model for Oct1 phosphorylation and ubiquitination through the cell cycle is shown in Figure 7. APC interacts with Oct1 and is required for Oct1 K11 ubiquitination, strongly suggesting that APC is the upstream Oct1 Ub ligase. We observe these interactions and activities outside of anaphase, however it is widely recognized that the APC is active throughout the cell cycle, including in early mitosis [33].

Although a simple model is that Oct1 is phosphorylated at S335 and becomes non-functional, several lines of evidence suggest that Oct1^pS335^ acquires new functions. In *Xenopus* egg extracts, Oct1 co-purifies with the spindle matrix, which helps maintain spindle shape. Oct1 also localizes with lamin B at the midbody. Lamin B1 and Oct1 are mutually required to localize each other to the midbody, suggesting that they form a complex. Aside from the specific localization to mitotic structures, results from both Oct1 loss- and gain-of-function experiments implicate Oct1 as a mitotic regulator, at least in some cell types. For example, the organization of the mitotic spindle is disrupted upon Oct1 siRNA knockdown in HeLa cells. In XEE, Oct1 depletion causes defects in spindle morphogenesis, implicating Oct1 in mitosis-specific functions. Based on the observed Oct1 localization patterns, it is likely that the mitotic irregularities caused by changes in Oct1 levels are directly associated with mitotic regulation. Furthermore, an intact serine at position 335 is important for the mitotic phenotype of over-expressed Oct1, implicating this residue in mitotic functions.

We found that Oct1 is not required for the completion of mitosis in HeLa cells but rather appears to play a regulatory role. In other cell types, such as murine fibroblasts and A549 cells, the effect of Oct1 is more mild than in HeLa cells. Oct1 deficient embryos survive past gastrulation [17], [34] and Oct1 deficient MEFs proliferate normally in culture [1], [17]. Primary Oct1 deficient MEFs undergo oncogenic transformation poorly relative to wild-type controls, but immortalize normally by serial passage [1]. In this sense, the role of Oct1 in mitosis may be more akin to BRCA1, which appears to act as a mitotic regulator rather than a core component of the mitotic machinery [25], [26], [27]. As a second example, lamin-B RNAi results in a delay in prometaphase, following which cells can finish mitosis [19].

## Methods

### Tissue Culture

Oct1 deficient MEFs have been described previously [17]. HeLa cells (ATCC) were arrested in M-phase using 0.5 µg/ml nocodozole for 18 hr. Thymidine block and release from nocodozole were performed identically to Matsumoto et al. [22]. MG132 (Calbiochem) was applied at 5 µM for 6 hr. For experiments using both nocodozole and MG132, cells were treated with nocodozole for 12 hr, following which MG132 was added at 5 µM and cells were incubated with nocodozole and MG132 for a further 6 hr. Cells were maintained in a humidified environment at 37°C with 5% CO_2_. HeLa cells were transiently transfected using polyethyleneimine (PEI, Sigma) and pCG-FLAG-Oct1 [35], [36] or S335 mutant Oct1 generated as described [10].

### Antibodies

A commercial rabbit phospho-specific antibody (Bethyl) was raised against the peptide EALNLS_335_FKNMC. The antibody was purified in two steps, first by blocking with unphosphorylated peptide to remove non-phospho-specific antibodies, then by affinity purification using the phosphorylated epitope. Mouse antibodies against α-tubulin, γ-tubulin, CLASP-1, lamin B, and goat anti-Pit-1, were obtained from Santa Cruz. Mouse anti-pS10-H3 was obtained from Cell Signaling, and mouse anti-glyceraldehyde 3-phosphate dehydrogenase (GAPDH) from Chemicon. Rabbit anti-APC1 and mouse anti-Cdh1 antibodies were purchased from Abcam. Mouse anti-FLAG and rabbit anti-Nek6 antibodies were from Sigma. For pan-Oct1, a rabbit antibody (Bethyl) was used with the exception of Figure 1D, which used a mouse antibody (Millipore). Rabbit anti-Ub antibodies were a gift of M. Rechsteiner. Human anti-K11-Ub antibodies were a gift of V. Dixit. Rabbit anti-Cdk11 was purchased from Bethyl.

### Spindle matrix preparation

Assembly of spindles from *Xenopus* egg extracts using AuroraA-conjugated beads was conducted as described [19]. For Oct1 immunodepletion, rabbit anti-Oct1 (Bethyl) or nonspecific IgG control (Sigma) antibodies were used similarly to Goodman et al. [37], except that 75 µl of magnetic protein A-Dynabead slurry was used per 100 µl of extract.

### Immunofluorescence

Coverslips were coated with Poly-L-lysine (Sigma) for 30 min and placed into 6 well dishes where counted cells were plated. Cells were washed with phosphate buffered saline (PBS) two times prior to fixation. Methanol fixation was performed as described [22]. For formaldehyde fixation, cells were incubated in 4% formaldehyde in CSK buffer (100 mM NaCl, 300 mM Sucrose and 10 mM PIPES pH6.8) for 30 min at room temperature (RT), and washed three times with CSK buffer plus protease inhibitor cocktail (PIs, Roche). Permeabilization was achieved by adding CSK buffer (+0.5% Triton-X-100 and PIs) for 10 min, followed by 3 washes with PBS-T (PBS+0.05% Tween-20). IF images using K11-Ub, pan-Ub, and pan-Oct1 antibodies used methanol fixation, while phospho-histone H3 antibodies used formaldehyde. Antibodies against CLASP-1, α- and γ-tubulin, lamin B and phospho-S335 worked efficiently with both fixation procedures. Fixed cells were blocked with IF buffer (PBS-T with 1% donkey serum) for 1 hr at RT. Primary and secondary antibodies diluted in the IF buffer and were incubated sequentially. After each incubation, cells were washed with PBS-T for 10 min. Stained coverslips were placed on slides using mounting medium with DAPI (Vector). Images were taken using a Zeiss Axioplan 2 imaging microscope with a 100× oil immersion objective and a numerical aperture of 1.3. Digital fluorescence and DIC images were acquired using an AxioCam MRm monochrome digital camera. Final images were processed, given false color and merged using Photoshop CS3 (Adobe Systems). All scale bars conform to 20 µM. Mitotic staging followed established criteria, e.g. Maiato et al. [38] and Pereira et al. [39].

### In Vitro Kinase Assay

Two sets of complimentary oligo DNA (S335 and A335) were anealed and ligated into pGEX-4T1 (Promega) using a *Sma*I restriction site. Expressed proteins in BL21 Codon-plus (Stratagene) *Escherichia coli* transformed with each construct were purified using glutathione-sepharose resin (GE Healthcare). Sequences were S335: 5′GCGATTTGAAGCCTTGAACCTCAGCTTTAAGAACATGTGCAAGTGA3′, 5′TCACTTGCACATGTTCTTAAAGCTGAGGTTCAAGGCTTCAAATCGC3′; A335: 5′GCGATTTGAAGCCTTGAACCTCGCCTTTAAGAACATGTGCAAGTGA3′, 5′TCACTTGCACATGTTCTTAAAGGCGAGGTTCAAGGCTTCAAATCGC3′. The GST-fused peptide (WT, RFEALNLS_335_FKNMCK or S335A) were incubated with recombinant Nek6 and CAK complex (cdk7/cyclin H/MAT1, Millipore). Kinase activities were assayed with the purified substrate according to a manufacturer's protocol.

### RNAi

siRNA pools targeting three different regions of Oct1 or Nek6 (Santa Cruz) were mixed with lipofectamine RNAi max (Invitrogen) and transiently transfected according to manufacturer's protocol. Control siRNAs were used in parallel and also purchased from Santa Cruz. Cells were cultured for 3 days prior to analysis. siRNA knockdown of CDH1 (gene symbol *Fzr1*) and control transfections used siRNA pools (Dharmacon). Cells were cultured for 48 hr prior to analysis.

### Immunoprecipitation

Cells were lysed with 18 mM Hepes pH 7.9, 150 mM NaCL, 1 mM EDTA, 1 mM EGTA, 1% Triton-X-100, protease inhibitors (Roche, PIs), and phosphatase inhibitors (Roche, PhIs). 500 µg of extract was incubated with 4 µg of antibody in IP buffer (50 mM Tris pH8.0, 20% glycerol, 0.5 mM EDTA, 0.1% NP-40, 1 mM DTT, PIs and PhIs) overnight at 4°C. Protein-antibody complexes were precipitated using magnetic beads (Activmotif) and washed three times with IP buffer.

## Supporting Information

Figure S1 **Further characterization of the phospho-specific antibody.** (**A**) The phospho-specific antibody immunoprecipitates ubiquitinated Oct1 under denaturing conditions. IP was performed as described in the materials and methods, except cells were lysed in the identical buffer but with 8 M urea. Upon dilution the resulting bead incubation step contained 1.6 M urea in buffer. (**B**) Immunofluorescence images are shown. Left and right panels show early and late mitosis, respectively. NT = no treatment. METHODS: samples were prepared as described in the materials and methods, except fixed samples were treated with CIP in CSK buffer at 30°C for 30 minutes.
(JPG)

Figure S2 **Localization of total Oct1 in Mitotic HeLa Cells.** (**A**) Total Oct1 concentrates at spindle pole bodies and the midbody, and is excluded from mitotic chromosomes. Immunofluorescence images are shown. HeLa cells were fixed and stained with antibodies against α-tubulin and pan-Oct1. Arrow indicates position of additional puncta not recognized by phospho-Oct1 antibodies. (**B**) Pan-Oct1 antibodies recognizing different epitopes stain different mitotic structures. Cells were fixed and stained as in (A). Arrows indicate additional metaphase puncta of unknown etiology not recognized by phospho-Oct1 antibodies. (C) Side-by-side images of normal interphase and mitotic HeLa cells stained with phospho-Oct1 or pan-Oct1 antibodies. Cells were fixed as in (A).
(JPG)

Figure S3 **Localization of Oct1 Phosphorylated at S335 to Mitotic A549 Lung Adenocarcinoma Cells.** (**A**) Immunofluorescence images are shown. Specific mitotic cell is highlighted with an arrow. The cells were co-stained with antibodies to alpha-tubulin and with DAPI. (**B**) Similar images using anti-gamma-tubulin antibodies. Protocols were identical to those summarized in the methods section.
(JPG)

Figure S4 **Comparison of the Properties of Anti-Oct1^p335^ and Anti-Histone H3^S10^ Antibodies.** (**A**) Low-magnification (200×) immunofluorescence images of HeLa cells stained with antibodies against phospho-histone H3 and phospho-Oct1^335^. Arrows indicate mitotic cells. White arrows show cells in earlier mitotic stages that co-stain with both antibodies. Yellow arrows show examples of cells staining only with the phospho-335 and not phospho-histone antibody. (**B**) Immunofluorescence images of mitotic HeLa cells stained with anti-Oct1^p335^ and anti-histone H3^S10^ antibodies. Scale bar: 20 µM.
(JPG)

Figure S5 **Effect of Oct1 siRNA knockdown on HeLa cells stained with alpha- and gamma-tubulin antibodies.** (A) Additional examples of mitotic abnormalities in HeLa cells treated with Oct1-specific siRNAs. Reference Fig. 3 for normal mitotic and scrambled siRNA controls. (B) Similar to (A) except anti-gamma tubulin antibodies were used. Mitotic examples are shown.
(JPG)

Figure S6 **Phospho-S335 IF Staining Pattern and Cell-cycle Phenotype of Primary MEFs.** (**A**) Immunofluorescence images of mitotic stages from wild-type primary early-passage MEFs. (**B**) Mitotic examples of *Oct1^−/−^* MEFs. These examples could not be easily staged due to abnormalities. Note the reduced pS335 staining. (**C**) Alignment of human Oct1 and mouse Oct1, Oct2 and Pit-1. Alignment was generated using a Clustal W-based algorithm within the Vector NTI software package. Lower panel shows a Western blot of HeLa cells, wild-type and Oct1 deficient fibroblasts. The Pit-1 antibody was obtained from Santa Cruz. (**D**) Cell cycle profile of primary early passage Oct1 deficient MEFs and wild-type littermate controls. Inset shows expanded view of cells with super-4N DNA content.
(JPG)

Figure S7 **Effect of WT and S335A Oct1 overexpression on Oct1^pS335^ in HeLa cells.** IF images are shown of interphase and HeLa cells transiently transfected with wild-type or S335A mutant pCG-Oct1.
(JPG)

Figure S8 **Ectopic Oct1 expression in HeLa cells increases alpha-tubulin levels, and induces formation of puncta containing alpha-tubulin but lacking DNA.** IF images are shown of interphase HeLa cells transiently transfected as in Figure S7. Fixed cells were stained with DAPI and antibodies against the FLAG epitope and a-tubulin. Scale bars indicate 20 µM.
(JPG)

Figure S9 **Additional evidence that Nek6 phosphorylates Oct1.** (**A**) Nek6 was knocked down in HeLa cells using siRNAs as in Fig. 4C, but using rabbit anti-pan-Oct1 antibodies. (**B**) Pan-Oct1 and phospho-Oct1 channel signal intensity was analyzed following Nek6 knockdown using ImageJ software (NIH). 6 control and 7 Nek6 siRNA mitotic events were averaged in the case of pan-Oct1, and 5 control and 5 Nek6 siRNA mitotic events were averaged in the case of phopsho-Oct1. (**C**) GST-fused to the substrate peptide was tested as an in vitro kinase target as in Fig. 4A but using γ-^32^P-ATP. (**D**) Full-length, His_6_-tagged recombinant Oct1 purified from *E. coli* was used. Right panel shows a Coomassie-stained gel of the same material. “Δ Oct1” indicates presumptive Oct1 deletion products that are also phosphorylated species. METHODS: Recombinant C-terminal His_6_-tag Oct1 was expressed and purified as described (Ström AC, *et al.*, *Nucleic Acids Res*. 1996 Jun 1;24(11):1981–6).
(JPG)

Figure S10 **Additional evidence of mutual localization mediated by lamin B1 and Oct1.** (**A**) HeLa cells were transiently transfected with siRNAs against lamin B1 (Santa Cruz), incubated for 72 hr and Western blotted with antibodies against lamin B1 or GAPDH as a loading control. (**B**) IF images are shown of mitotic HeLa cells transiently transfected with control or lamin B1 siRNAs, and stained with antibodies against lamin B (B1+B2) and Oct1^pS335^ antibodies. Arrows highlight elongated/split spindle poles.
(JPG)

Figure S11 **APC is responsible for the accumulation of K11-Ub signal at sites of Oct1 phosphorylation upon proteasome inhibition.** HeLa cells were transiently transfected with control siRNAs or siRNAs directed against the APC component CDH1 (gene symbol *Fzr1*, Dharmacon), incubated for 48 hr. After 42 hr, cells were treated with MG132 as described in the materials section. Cells were then fixed and processed for IF. For quantification, mitotic events were scored +/− based on concordance between Oct1^pS335^ and K11-Ub staining.
(JPG)
