# Supplementary figures and images for: Dynamic Regulation of Oct1 during Mitosis by Phosphorylation and Ubiquitination
Source: PLoS One. 2011 Aug 29;6(8):e23872. doi: 10.1371/journal.pone.0023872 (PMC3163677; doi:10.1371/journal.pone.0023872)

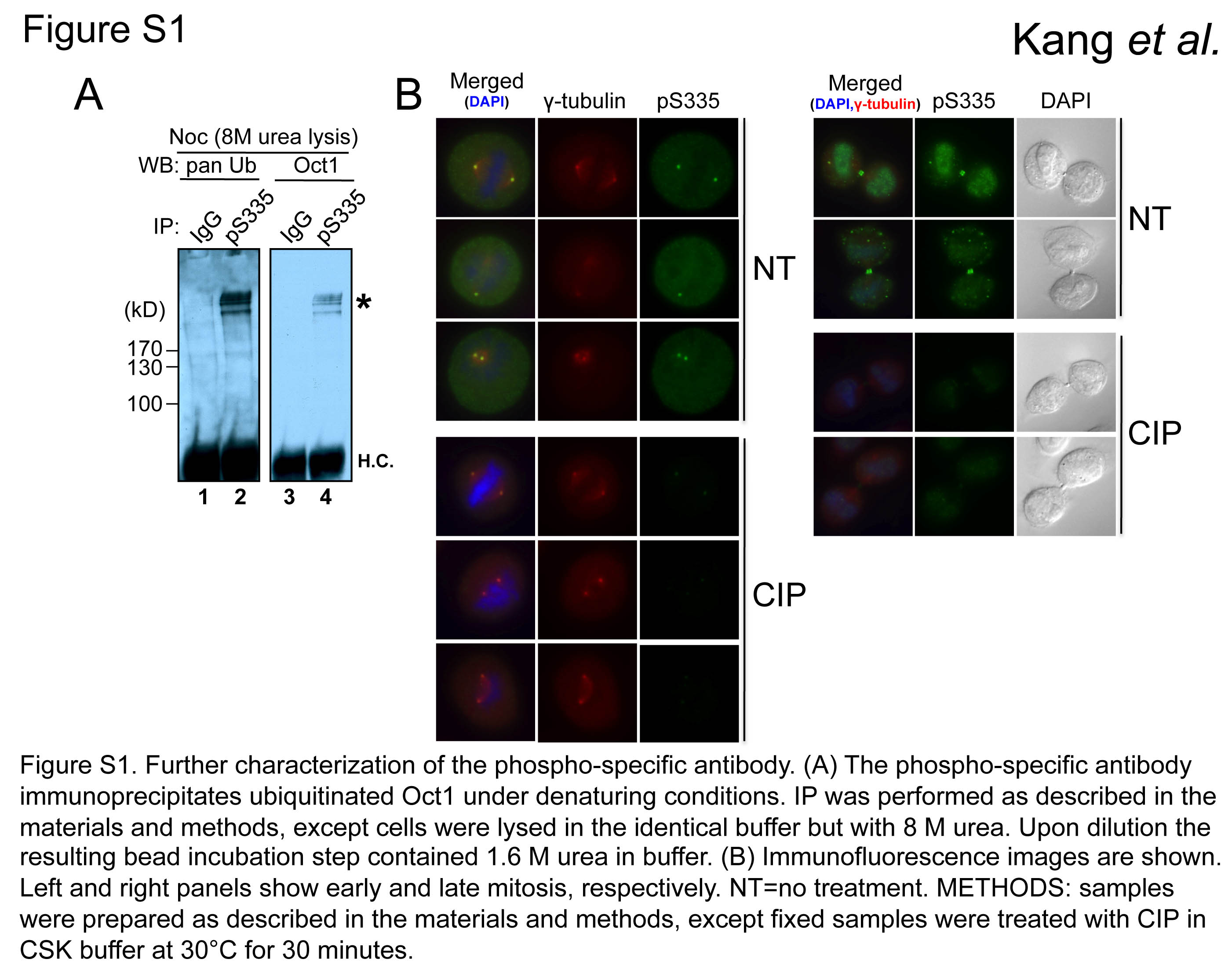

Supplement: Figure S1 — Further characterization of the phospho-specific antibody. (A) The phospho-specific antibody immunoprecipitates ubiquitinated Oct1 under denaturing conditions. IP was performed as described in the materials and methods, except cells were lysed in the identical buffer but with 8 M urea. Upon dilution the resulting bead incubation step contained 1.6 M urea in buffer. (B) Immunofluorescence images are shown. Left and right panels show early and late mitosis, respectively. NT = no treatment. METHODS: samples were prepared as described in the materials and methods, except fixed samples were treated with CIP in CSK buffer at 30°C for 30 minutes. (JPG) [file pone.0023872.s001.jpg]

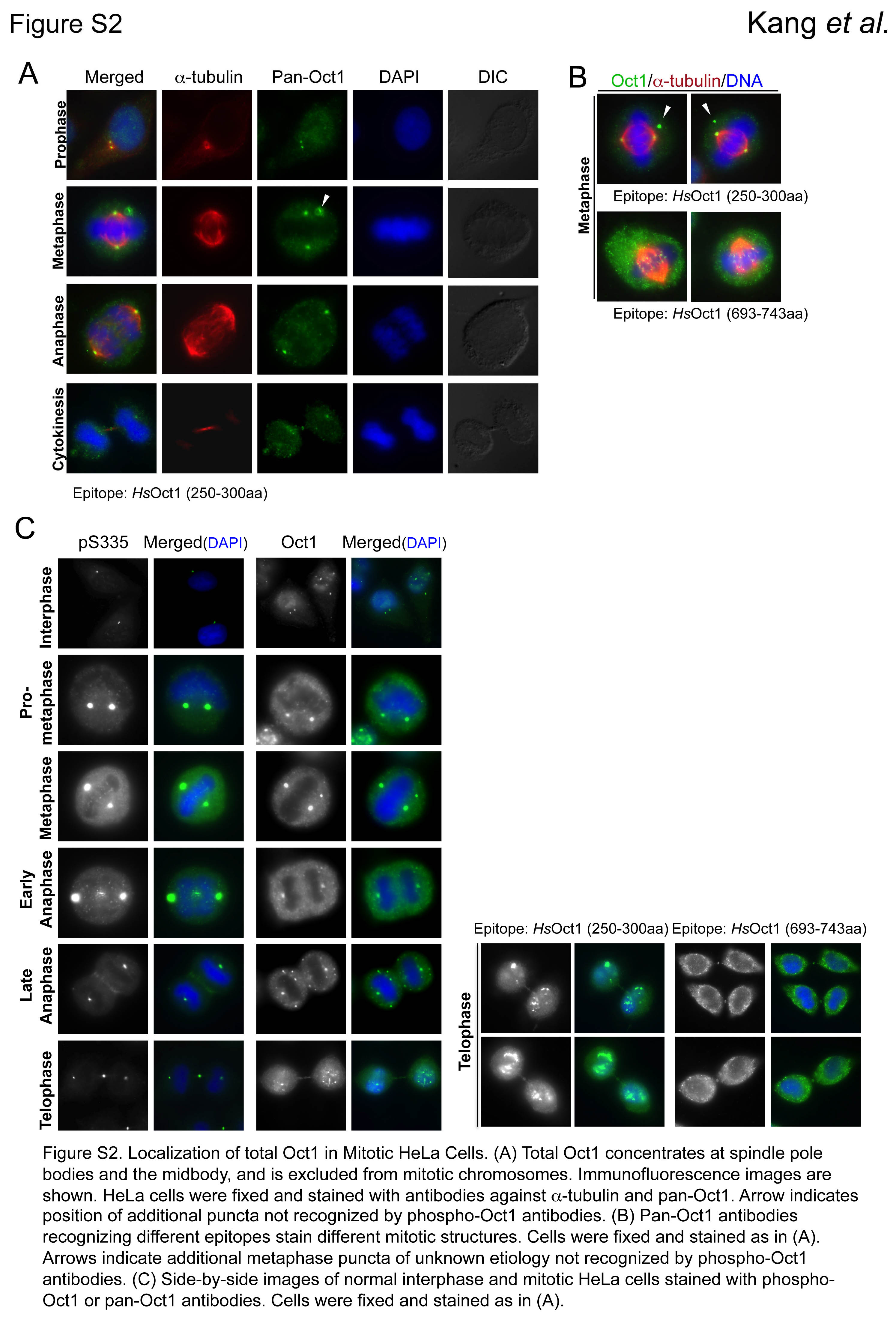

Supplement: Figure S2 — Localization of total Oct1 in Mitotic HeLa Cells. (A) Total Oct1 concentrates at spindle pole bodies and the midbody, and is excluded from mitotic chromosomes. Immunofluorescence images are shown. HeLa cells were fixed and stained with antibodies against α-tubulin and pan-Oct1. Arrow indicates position of additional puncta not recognized by phospho-Oct1 antibodies. (B) Pan-Oct1 antibodies recognizing different epitopes stain different mitotic structures. Cells were fixed and stained as in (A). Arrows indicate additional metaphase puncta of unknown etiology not recognized by phospho-Oct1 antibodies. (C) Side-by-side images of normal interphase and mitotic HeLa cells stained with phospho-Oct1 or pan-Oct1 antibodies. Cells were fixed as in (A). (JPG) [file pone.0023872.s002.jpg]

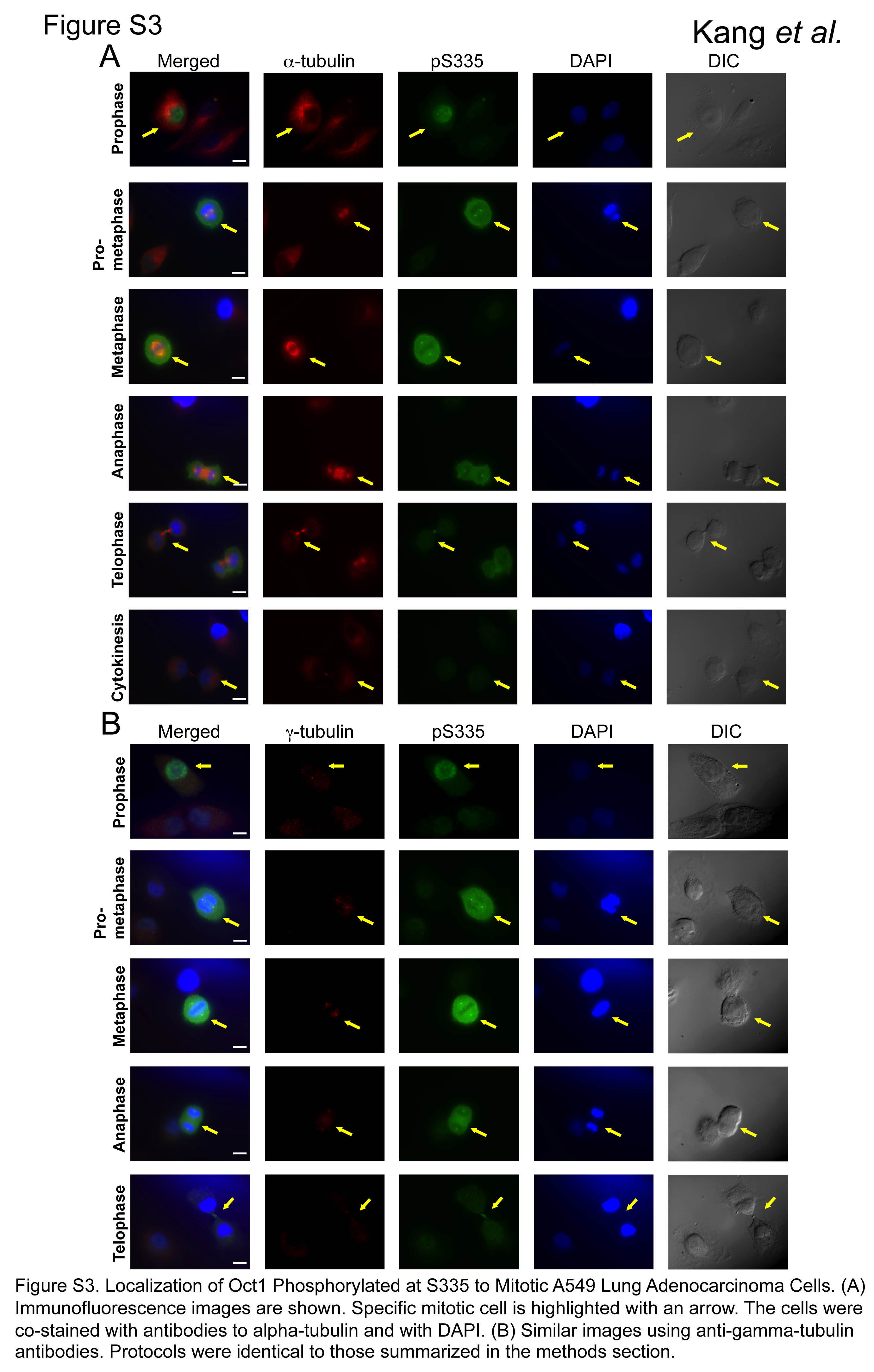

Supplement: Figure S3 — Localization of Oct1 Phosphorylated at S335 to Mitotic A549 Lung Adenocarcinoma Cells. (A) Immunofluorescence images are shown. Specific mitotic cell is highlighted with an arrow. The cells were co-stained with antibodies to alpha-tubulin and with DAPI. (B) Similar images using anti-gamma-tubulin antibodies. Protocols were identical to those summarized in the methods section. (JPG) [file pone.0023872.s003.jpg]

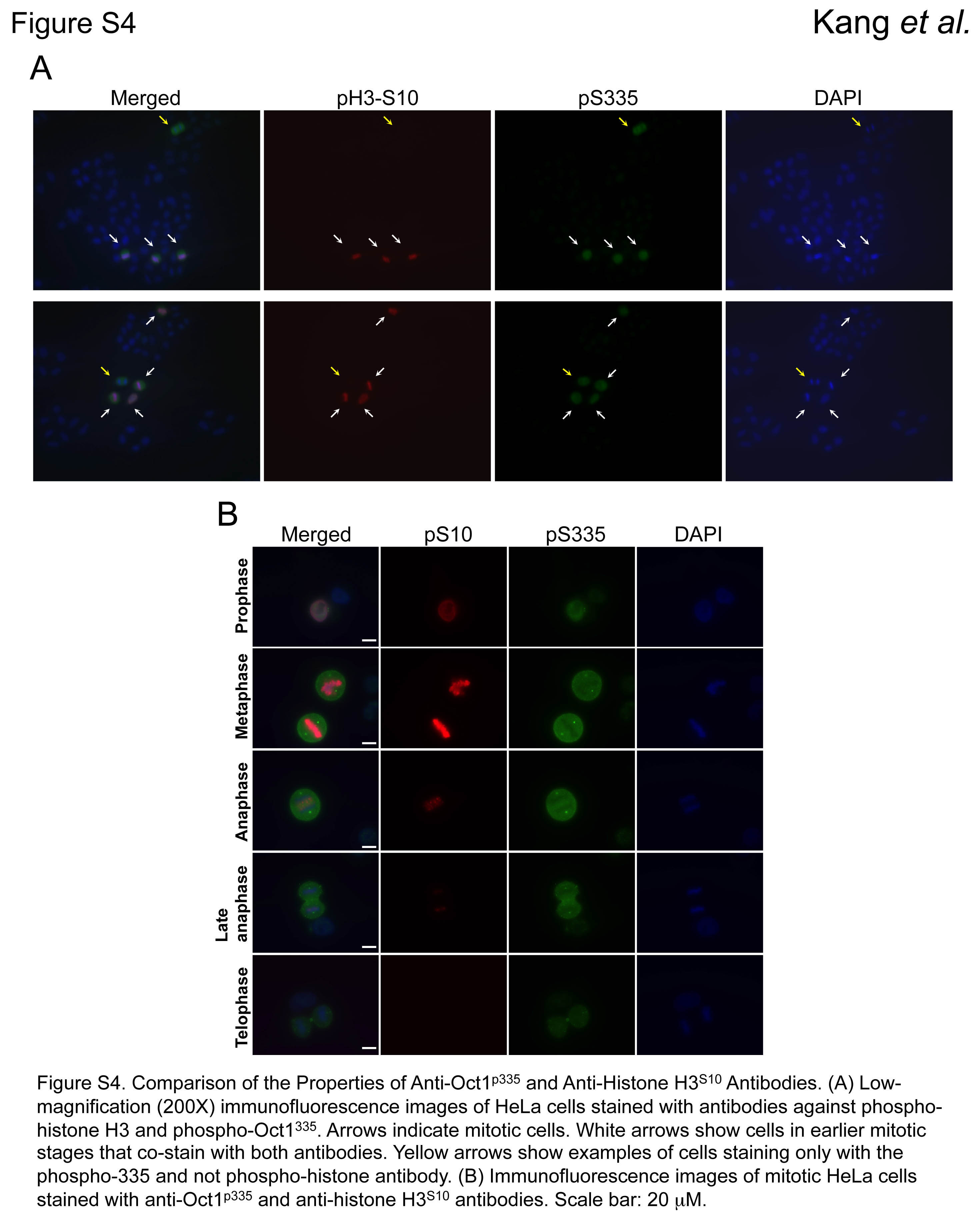

Supplement: Figure S4 — Comparison of the Properties of Anti-Oct1p335 and Anti-Histone H3S10 Antibodies. (A) Low-magnification (200×) immunofluorescence images of HeLa cells stained with antibodies against phospho-histone H3 and phospho-Oct1335. Arrows indicate mitotic cells. White arrows show cells in earlier mitotic stages that co-stain with both antibodies. Yellow arrows show examples of cells staining only with the phospho-335 and not phospho-histone antibody. (B) Immunofluorescence images of mitotic HeLa cells stained with anti-Oct1p335 and anti-histone H3S10 antibodies. Scale bar: 20 µM. (JPG) [file pone.0023872.s004.jpg]

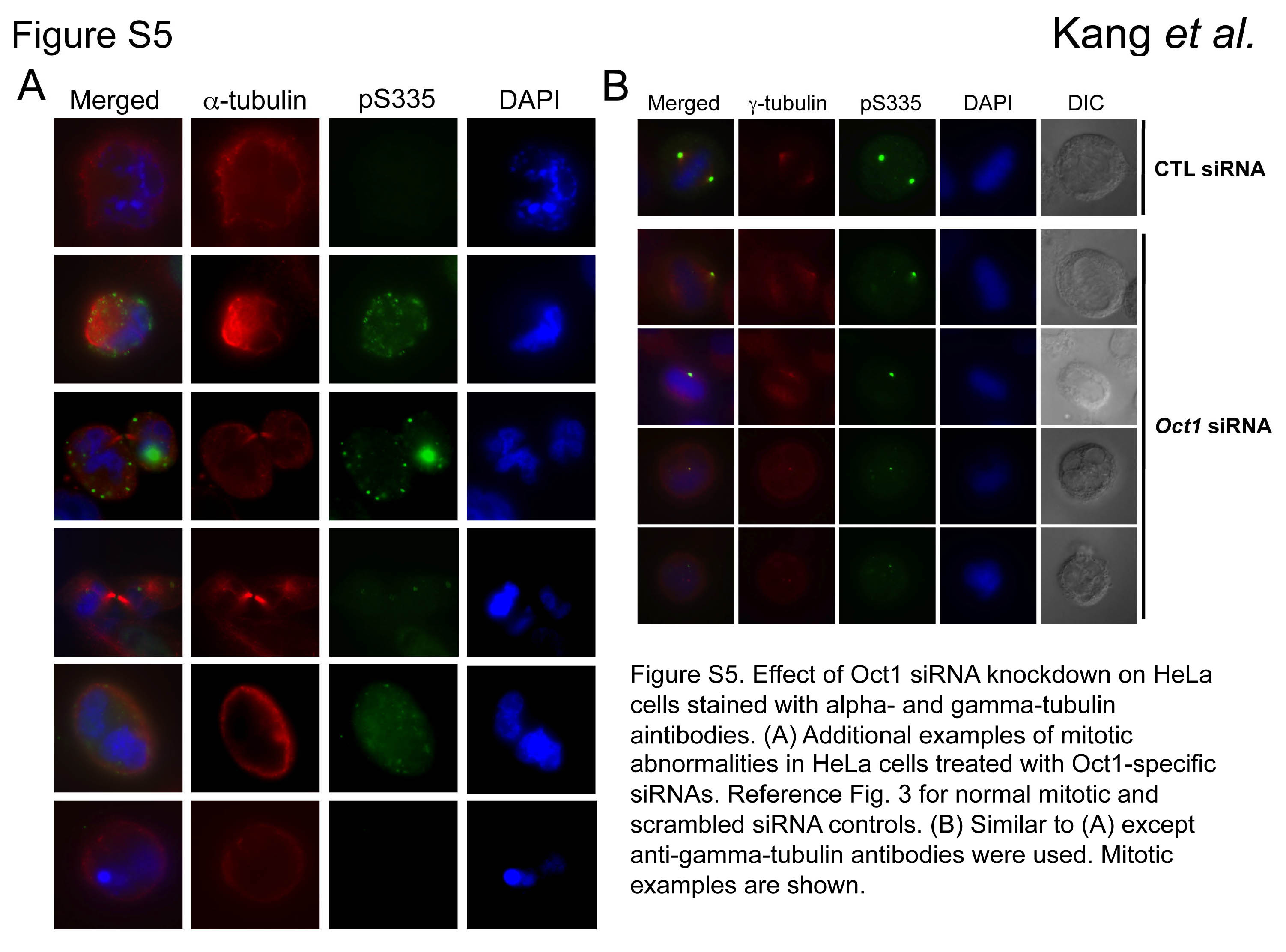

Supplement: Figure S5 — Effect of Oct1 siRNA knockdown on HeLa cells stained with alpha- and gamma-tubulin antibodies. (A) Additional examples of mitotic abnormalities in HeLa cells treated with Oct1-specific siRNAs. Reference Fig. 3 for normal mitotic and scrambled siRNA controls. (B) Similar to (A) except anti-gamma tubulin antibodies were used. Mitotic examples are shown. (JPG) [file pone.0023872.s005.jpg]

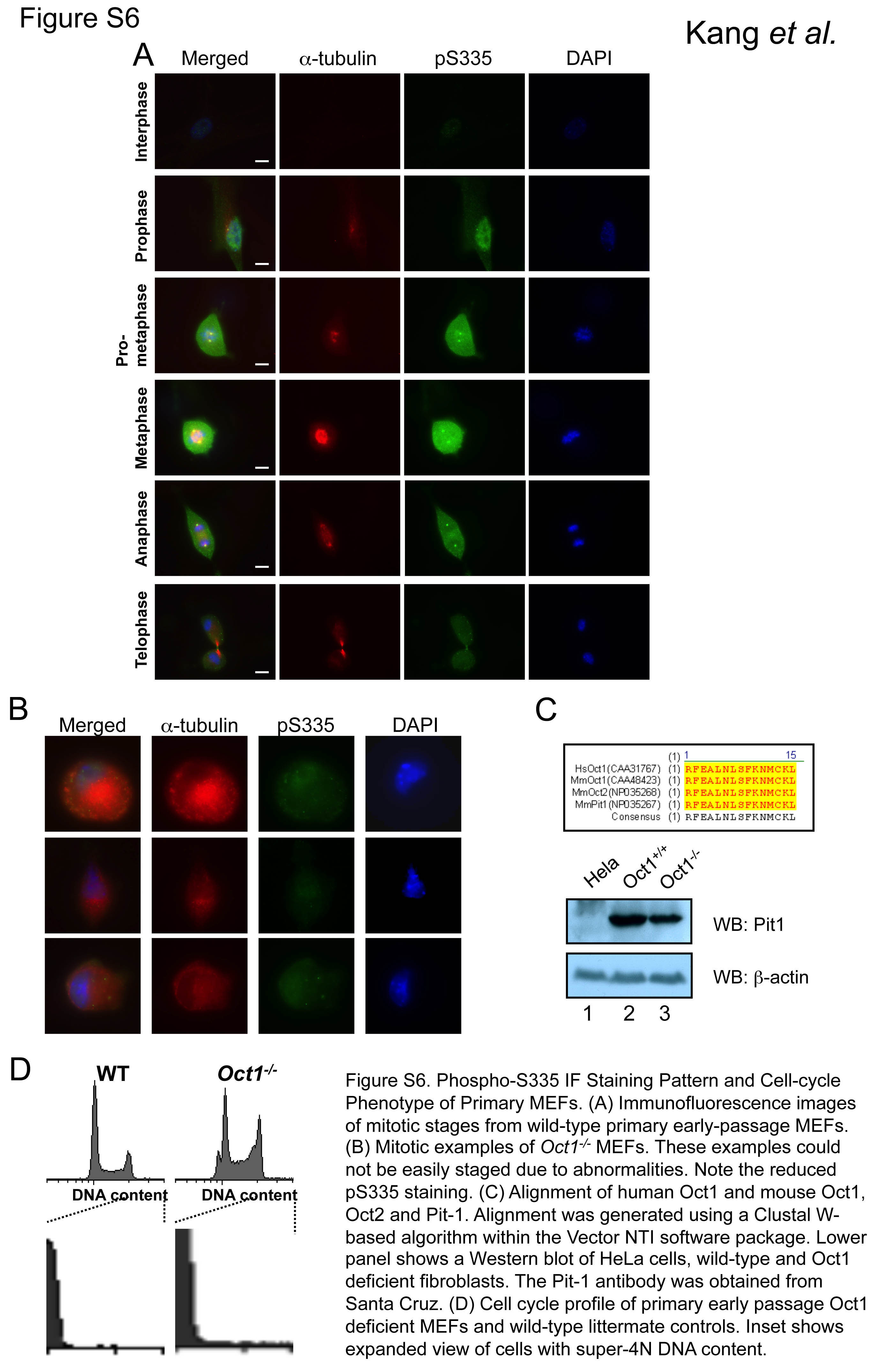

Supplement: Figure S6 — Phospho-S335 IF Staining Pattern and Cell-cycle Phenotype of Primary MEFs. (A) Immunofluorescence images of mitotic stages from wild-type primary early-passage MEFs. (B) Mitotic examples of Oct1−/− MEFs. These examples could not be easily staged due to abnormalities. Note the reduced pS335 staining. (C) Alignment of human Oct1 and mouse Oct1, Oct2 and Pit-1. Alignment was generated using a Clustal W-based algorithm within the Vector NTI software package. Lower panel shows a Western blot of HeLa cells, wild-type and Oct1 deficient fibroblasts. The Pit-1 antibody was obtained from Santa Cruz. (D) Cell cycle profile of primary early passage Oct1 deficient MEFs and wild-type littermate controls. Inset shows expanded view of cells with super-4N DNA content. (JPG) [file pone.0023872.s006.jpg]

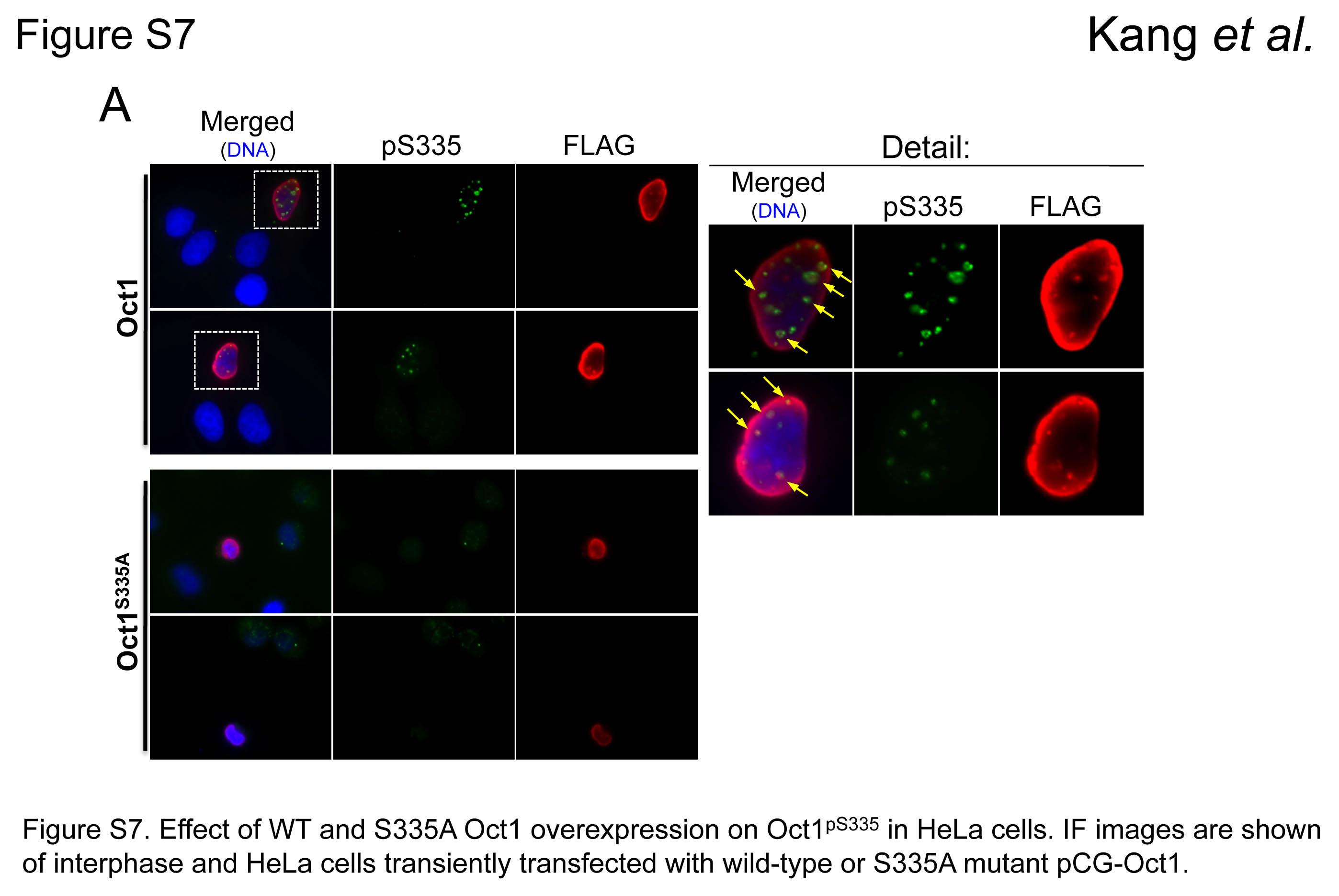

Supplement: Figure S7 — Effect of WT and S335A Oct1 overexpression on Oct1pS335 in HeLa cells. IF images are shown of interphase and HeLa cells transiently transfected with wild-type or S335A mutant pCG-Oct1. (JPG) [file pone.0023872.s007.jpg]

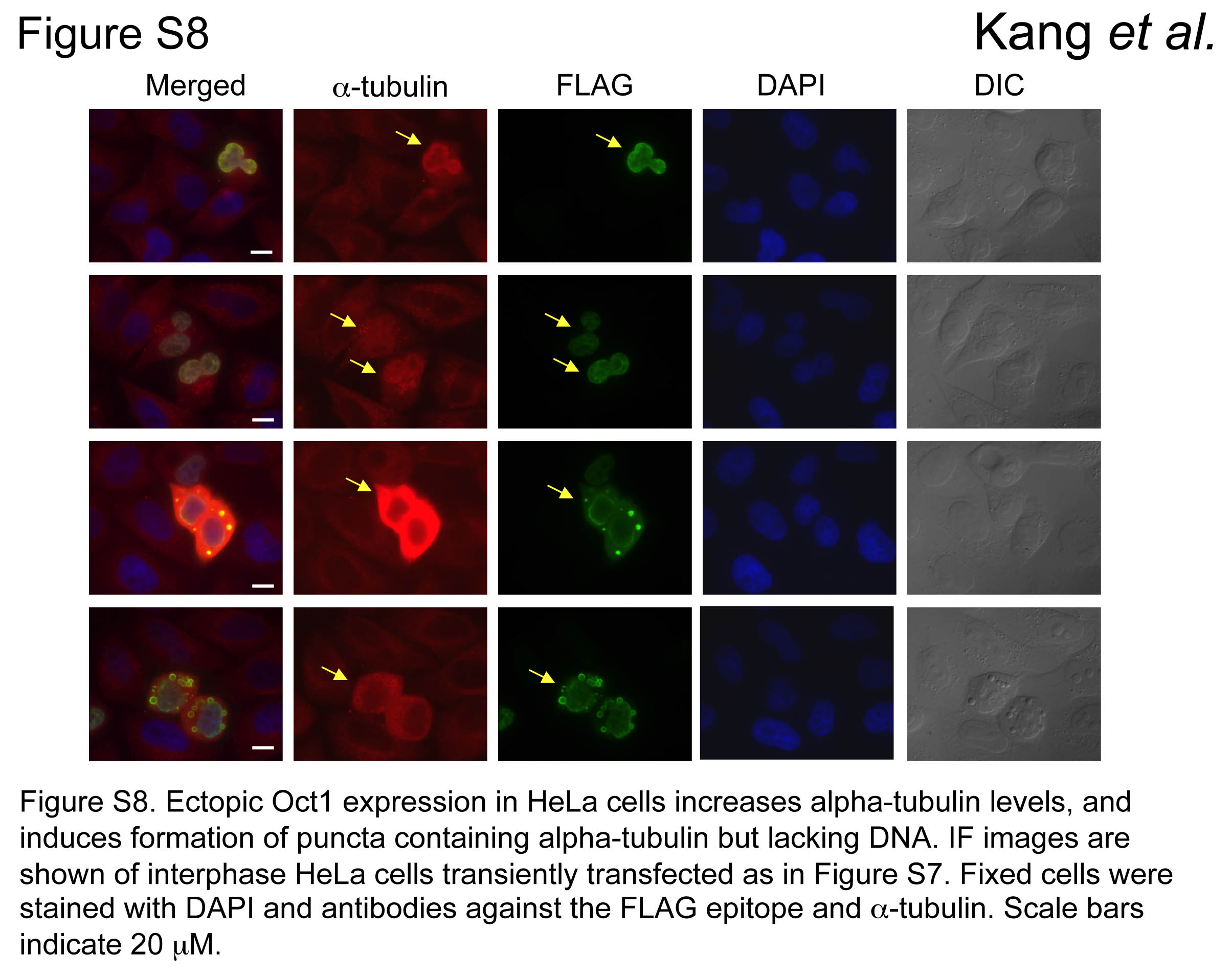

Supplement: Figure S8 — Ectopic Oct1 expression in HeLa cells increases alpha-tubulin levels, and induces formation of puncta containing alpha-tubulin but lacking DNA. IF images are shown of interphase HeLa cells transiently transfected as in Figure S7. Fixed cells were stained with DAPI and antibodies against the FLAG epitope and a-tubulin. Scale bars indicate 20 µM. (JPG) [file pone.0023872.s008.jpg]

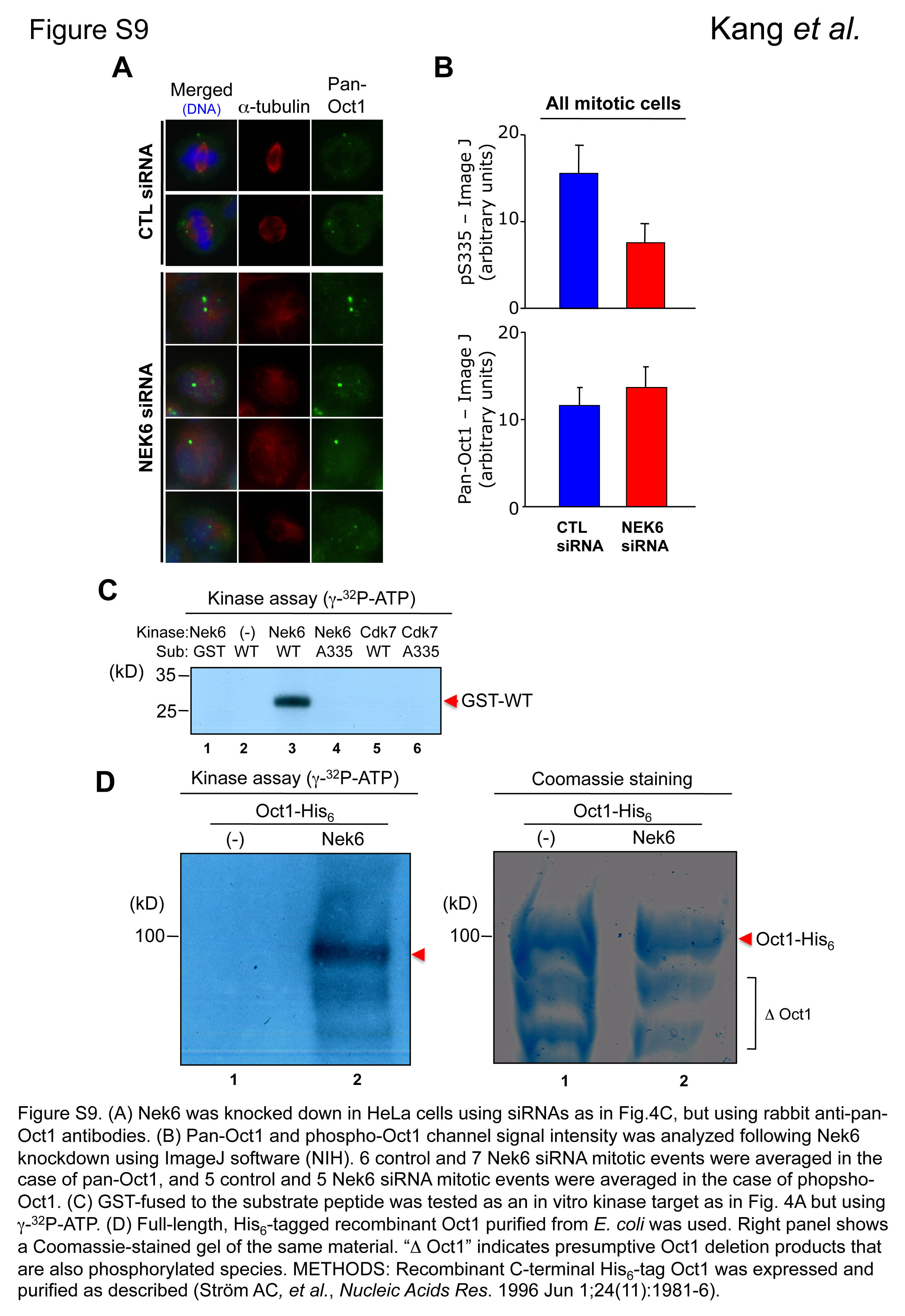

Supplement: Figure S9 — Additional evidence that Nek6 phosphorylates Oct1. (A) Nek6 was knocked down in HeLa cells using siRNAs as in Fig. 4C, but using rabbit anti-pan-Oct1 antibodies. (B) Pan-Oct1 and phospho-Oct1 channel signal intensity was analyzed following Nek6 knockdown using ImageJ software (NIH). 6 control and 7 Nek6 siRNA mitotic events were averaged in the case of pan-Oct1, and 5 control and 5 Nek6 siRNA mitotic events were averaged in the case of phopsho-Oct1. (C) GST-fused to the substrate peptide was tested as an in vitro kinase target as in Fig. 4A but using γ-32P-ATP. (D) Full-length, His6-tagged recombinant Oct1 purified from E. coli was used. Right panel shows a Coomassie-stained gel of the same material. “Δ Oct1” indicates presumptive Oct1 deletion products that are also phosphorylated species. METHODS: Recombinant C-terminal His6-tag Oct1 was expressed and purified as described (Ström AC, et al., Nucleic Acids Res. 1996 Jun 1;24(11):1981–6). (JPG) [file pone.0023872.s009.jpg]

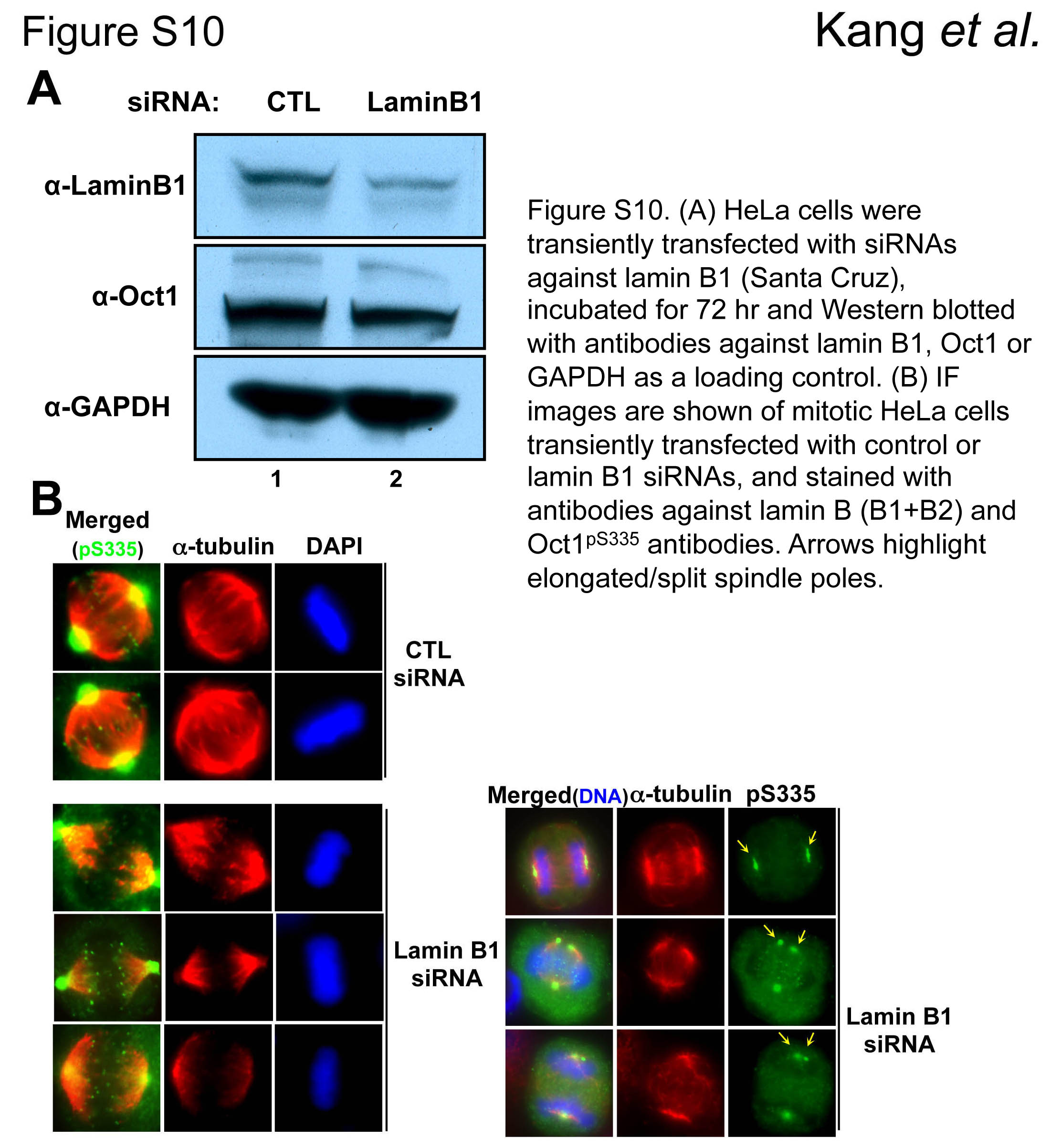

Supplement: Figure S10 — Additional evidence of mutual localization mediated by lamin B1 and Oct1. (A) HeLa cells were transiently transfected with siRNAs against lamin B1 (Santa Cruz), incubated for 72 hr and Western blotted with antibodies against lamin B1 or GAPDH as a loading control. (B) IF images are shown of mitotic HeLa cells transiently transfected with control or lamin B1 siRNAs, and stained with antibodies against lamin B (B1+B2) and Oct1pS335 antibodies. Arrows highlight elongated/split spindle poles. (JPG) [file pone.0023872.s010.jpg]

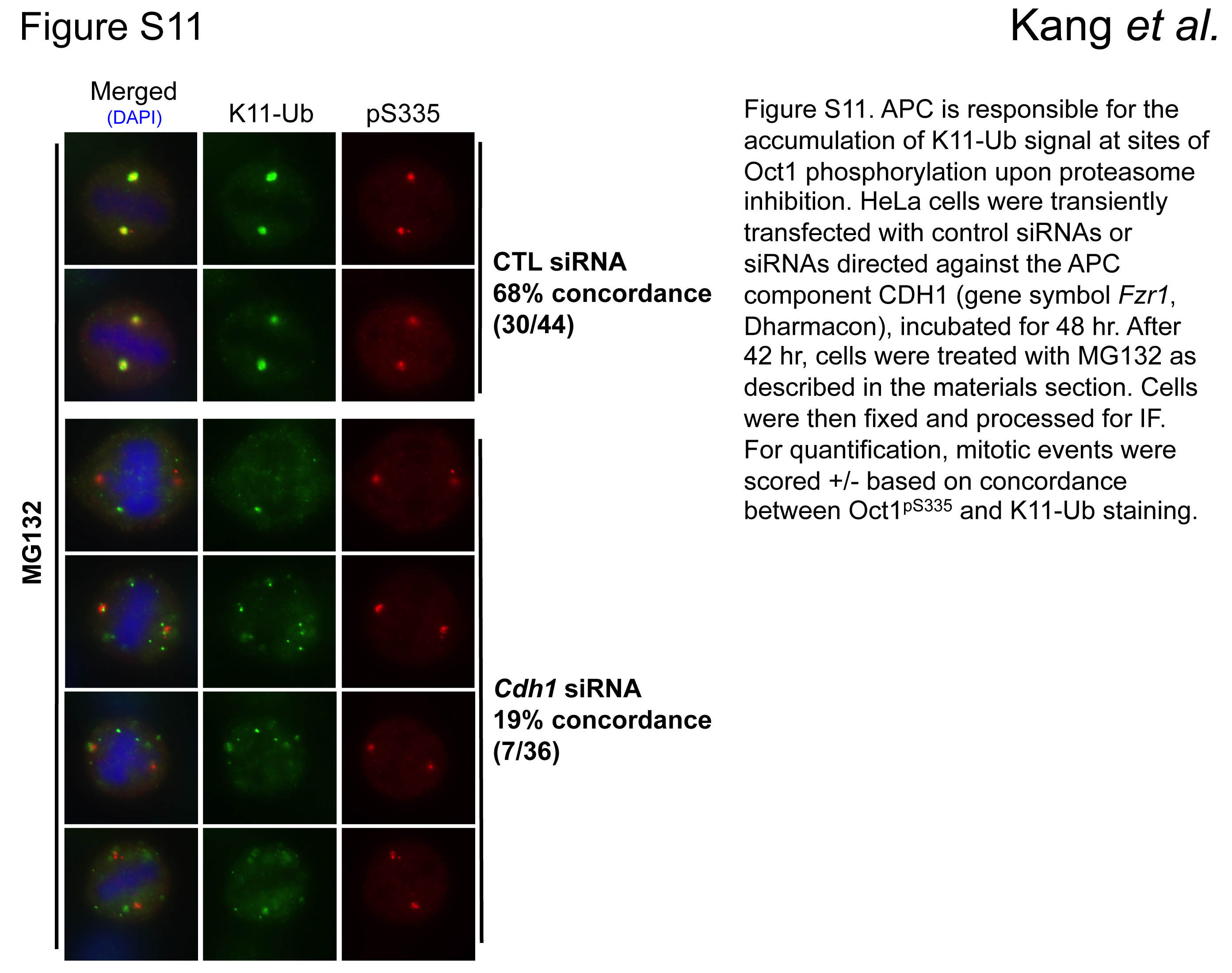

Supplement: Figure S11 — APC is responsible for the accumulation of K11-Ub signal at sites of Oct1 phosphorylation upon proteasome inhibition. HeLa cells were transiently transfected with control siRNAs or siRNAs directed against the APC component CDH1 (gene symbol Fzr1, Dharmacon), incubated for 48 hr. After 42 hr, cells were treated with MG132 as described in the materials section. Cells were then fixed and processed for IF. For quantification, mitotic events were scored +/− based on concordance between Oct1pS335 and K11-Ub staining. (JPG) [file pone.0023872.s011.jpg]
